# Activation of ATP Consumption Is Necessary to Stimulate Glycogenolysis in Skeletal Muscle

**DOI:** 10.3390/ijms27167106

**Published:** 2026-08-08

**Authors:** Michael V. Martinov, Svetlana I. Sudarkina, Fazoil I. Ataullakhanov, Victor M. Vitvitsky

**Affiliations:** 1 Center for Theoretical Problems of Physico-Chemical Pharmacology, Russian Academy of Sciences, Moscow 109029, Russia; martinov.michael@gmail.com (M.V.M.);; 2 Faculty of Computer Sciences, National Research University Highest School of Economics, Moscow 109028, Russia

**Keywords:** skeletal muscle, energy metabolism, glycolysis, glycogen, glycogen phosphorylase, ATP, mathematical model

## Abstract

Glycogenolysis is an important contributor to ATP production in contracting skeletal muscle. However, the activation of glycogen phosphorylase in resting muscle does not by itself trigger significant glycogenolysis. To elucidate the mechanisms underlying this regulation, we analyzed the functional coupling between glycogenolysis and glycolysis using a mathematical model of energy metabolism in mammalian fast-twitch (white) skeletal muscle. A system-level analysis of the model reveals an important role for inorganic phosphate (a substrate for glycogen phosphorylase) in regulating glycogenolysis rates in both resting and contracting muscle. In contracting muscle, at sufficiently high concentrations of inorganic phosphate, the activation of glycogen phosphorylase markedly increases the rate of glycogenolysis, thereby enhancing ATP production and stabilizing the cellular energy charge. In contrast, glycogen phosphorylase activation in resting muscle is unable to support glycogen breakdown due to a significant decrease in inorganic phosphate levels. Moreover, the analysis shows that forced acceleration of the glycogen phosphorylase reaction under resting conditions does not increase ATP production; instead, it promotes the accumulation of phosphorylated glycolytic intermediates, which may induce osmotic stress and cause damage to muscle cells.

## 1. Introduction

An important component of energy metabolism in different mammalian cells is glycogen metabolism. While liver glycogen plays a key role in maintaining blood glucose homeostasis and supporting whole-body energy metabolism, glycogen in muscle cells serves as an energy source exclusively within these cells [1,2,3,4,5]. The role of glycogen is most pronounced under stressful conditions in so-called white (fast-twitch) skeletal muscle, where it acts as the primary substrate for ATP production via glycolysis [5,6,7,8,9]. Despite decades of intensive research, the mechanisms regulating glycogenolysis in skeletal muscle remain incompletely understood [4].

The principal enzyme regulating glycogen consumption for energy production is glycogen phosphorylase (GP). This enzyme exists in phosphorylated and dephosphorylated forms. The dephosphorylated form (GPb) is allosterically activated by AMP but exhibits very low catalytic activity under physiological conditions, whereas the phosphorylated form (GPa) is highly active and largely insensitive to allosteric regulation [4,5,10,11,12,13,14,15,16]. The phosphorylation of GP can be stimulated by nerve impulses via Ca^2+^ ions released from the sarcoplasmic reticulum into the cytoplasm, as well as by hormones, including adrenaline, via the cAMP–protein kinase A–phosphorylase kinase signaling pathway [5,17]. Data obtained with purified GP demonstrate that, under intracellular conditions, the reaction rate catalyzed by both GPb and GPa increases with increasing concentrations of orthophosphate, which serves as a substrate for the enzyme [4,10,14,18]. In contracting muscle, the rate of glycogenolysis is proportional to the exercise intensity (i.e., the rate of ATP consumption) and is associated with increased concentrations of orthophosphate and AMP, as well as with the conversion of GPb to GPa [4,18,19,20,21,22,23]. In contrast, it has been shown that none of these factors—elevated orthophosphate or AMP concentrations or GPb-to-GPa conversion induced by adrenaline infusion—significantly stimulates glycogenolysis in resting muscle [4,18,19,22,24,25]. Based on these observations, it has been suggested that additional contraction-associated factors, distinct from orthophosphate and AMP concentrations or GPa levels, stimulate glycogenolysis in contracting muscle and remain to be identified [4,18,22,24].

To better understand the interaction between glycolysis and glycogenolysis in mammalian white skeletal muscle, a systems analysis approach based on the mathematical modeling of muscle energy metabolism can be employed. Attempts to apply systems analysis to the regulation of glycogen metabolism and its interaction with glycolysis have been made for more than 50 years [26,27,28,29,30,31,32]. However, only a few studies have considered models that explicitly include the interaction between glycolysis and glycogenolysis in mammalian skeletal muscle [28,29,30,31]. Moreover, none of these models addresses the question of why the activation of GP does not lead to the activation of glycogenolysis in resting skeletal muscle.

In addition, these studies generally do not examine the steady-state characteristics of muscle energy metabolism, including the steady-state dependence of the rate of glycolysis on the ATP concentration, which determines both the maximum rate of ATP production by glycolysis and the effectiveness of stabilizing intracellular ATP levels [33,34,35,36]. This represents a significant limitation, since muscle energy metabolism must operate at or near steady-state conditions. Indeed, the rate of ATP consumption in stimulated skeletal muscle can reach approximately 3 mmol·s^−1^·kg^−1^ of tissue [21,22,37,38]. Given the ATP concentration in skeletal muscle is approximately 5 mmol·kg^−1^ of tissue [9,37,38,39,40,41,42,43,44], intracellular ATP could be depleted within only a few seconds if the rate of ATP production does not closely match the rate of ATP consumption.

Thus, the rates of ATP consumption and production in muscle should be equal or very close to each other. This, in turn, ensures the constancy (stabilization) of the ATP concentration with changes in the rate of ATP consumption. These conditions—the equality of the rates of production and consumption of the metabolite and the constancy of its concentration—are in fact the conditions of a steady state. Given the high ATP turnover rates and the relatively low ATP concentration in muscles, it can be concluded that deviations from the steady state in skeletal muscle energy metabolism should not be large and long-lasting.

**Figure 1:**
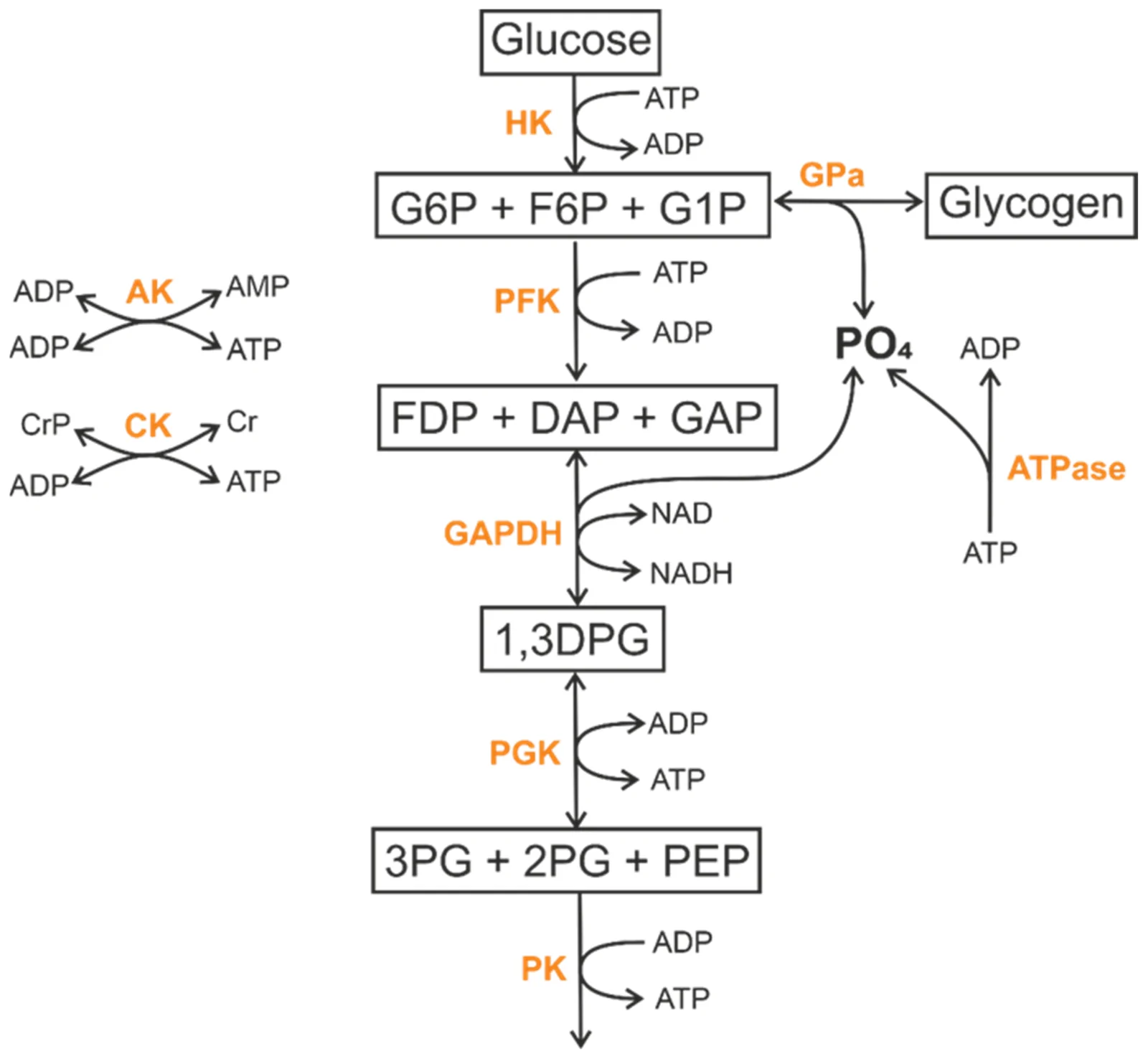
Enzymatic reactions included in the model. Metabolite sums enclosed in rectangular boxes represent pools corresponding to equilibrium enzymatic reactions. Concentrations of individual metabolites within each pool are determined by the equilibrium constants of the corresponding reactions. Abbreviations are defined in Table 1. The increased size of the orthophosphate symbol emphasizes the important role of orthophosphate in regulating the rate of glycogenolysis.

In this work, we analyze in greater detail the effects of glycogenolysis on energy metabolism in white (anaerobic) skeletal muscle, with a particular emphasis on the steady-state metabolic characteristics of the muscle’s energy metabolism. To this end, we employed a simple qualitative mathematical model of muscle energy metabolism. The model includes reaction rate equations for GPa, hexokinase (HK), phosphofructokinase (PFK), glyceraldehyde-3-phosphate dehydrogenase (GAPDH), phosphoglycerate kinase (PGK), and pyruvate kinase (PK) (Figure 1). Experimental data show that skeletal muscle consumes blood glucose via HK at rest and even during contraction, although the contribution of glucose to ATP production in contracting muscles is relatively small [6,45,46]. Because of this, and because we used glycolysis in resting skeletal muscle as a reference state in the model, HK was therefore included in the model. Experimental values of enzyme kinetic parameters were used, and key regulatory features were incorporated, including the inhibition of HK by glucose-6-phosphate (G6P) and the inhibition of PFK by ATP, together with its activation by AMP. The remaining glycolytic reactions, as well as the phosphoglucomutase (PGLM) reaction, were represented by the corresponding metabolite equilibrium relationships and stoichiometric coefficients. The model also includes equilibria for creatine kinase (CK) and adenylate kinase (AK) reactions. The combined activity of ATP-consuming processes is represented in the model as ATPase activity. The contribution of oxidative phosphorylation to ATP production was neglected, as it is minimal during contraction of white (anaerobic) skeletal muscle [7,8,21,45,47].

**Table 1:** List of abbreviations.

Metabolite		Enzyme/Reaction	
1,3DPG	1,3-Diphosphoglycerate	AK	Adenylate kinase
2PG	2-Phosphoglycerate	ALD	Aldolase
3PG	3-Phosphoglycerate	ATPase	Sum of ATP-consuming processes
Cr	Creatine	CK	Creatine kinase
CrP	Creatine phosphate	ENO	Enolase
DAP	Dihydroxyacetone phosphate	GAPDH	Glyceraldehyde 3-phosphate dehydrogenase
F6P	Fructose 6-phosphate	GP	Glycogen phosphorylase
FDP	Fructose diphosphate	GPa	Glycogen phosphorylase a
G1P	Glucose 1-phosphate	GPb	Glycogen phosphorylase b
G6P	Glucose 6-phosphate	GPI	Glucose-6-phosphate isomerase
GAP	Glyceraldehyde phosphate	HK	Hexokinase
PEP	Phosphoenolpyruvate	PFK	Phosphofructokinase
PO_4_	Free inorganic phosphate (orthophosphate)	PGK	Phosphoglycerate kinase
PYR	Pyruvate	PGM	Phosphoglycerate mutase
		PGLM	Phosphoglucomutase
		PK	Pyruvate kinase
		TPI	Triosephosphate isomerase

Using this model, we investigated the effects of GPa activation on skeletal muscle energy metabolism under steady-state conditions as well as during transient processes. Our results show that the orthophosphate concentration plays a key role in the regulation of energy metabolism in skeletal muscle. In muscle cells, the orthophosphate concentration is determined by the balance between its production in the contractile system and other ATP-consuming processes and its consumption in GP and CK reactions. Model analysis demonstrates that the steady-state orthophosphate concentration increases with increasing contractile activity. Under these conditions, the activation of GPa leads to an increase in the steady-state rate of glycogenolysis that is proportional to the orthophosphate concentration. In contrast, the activation of GPa in resting skeletal muscle cannot sustain steady glycogenolysis due to the rapid depletion of orthophosphate. The regulation of glycogenolysis via orthophosphate enables muscle cells to efficiently match ATP production to contractile activity. Thus, there is no need to invoke unknown contraction-associated factors to explain the regulation of glycogenolysis in skeletal muscle [4,18,22,24].

Finally, the model analysis shows that the forced acceleration of the GP reaction in resting skeletal muscle does not increase ATP production but instead promotes the accumulation of phosphorylated glycolytic intermediates, which may induce osmotic stress and damage muscle cells.

## 2. Results

### 2.1. Steady-State Characteristics of Muscle Energy Metabolism at Rest

**Figure 2:**
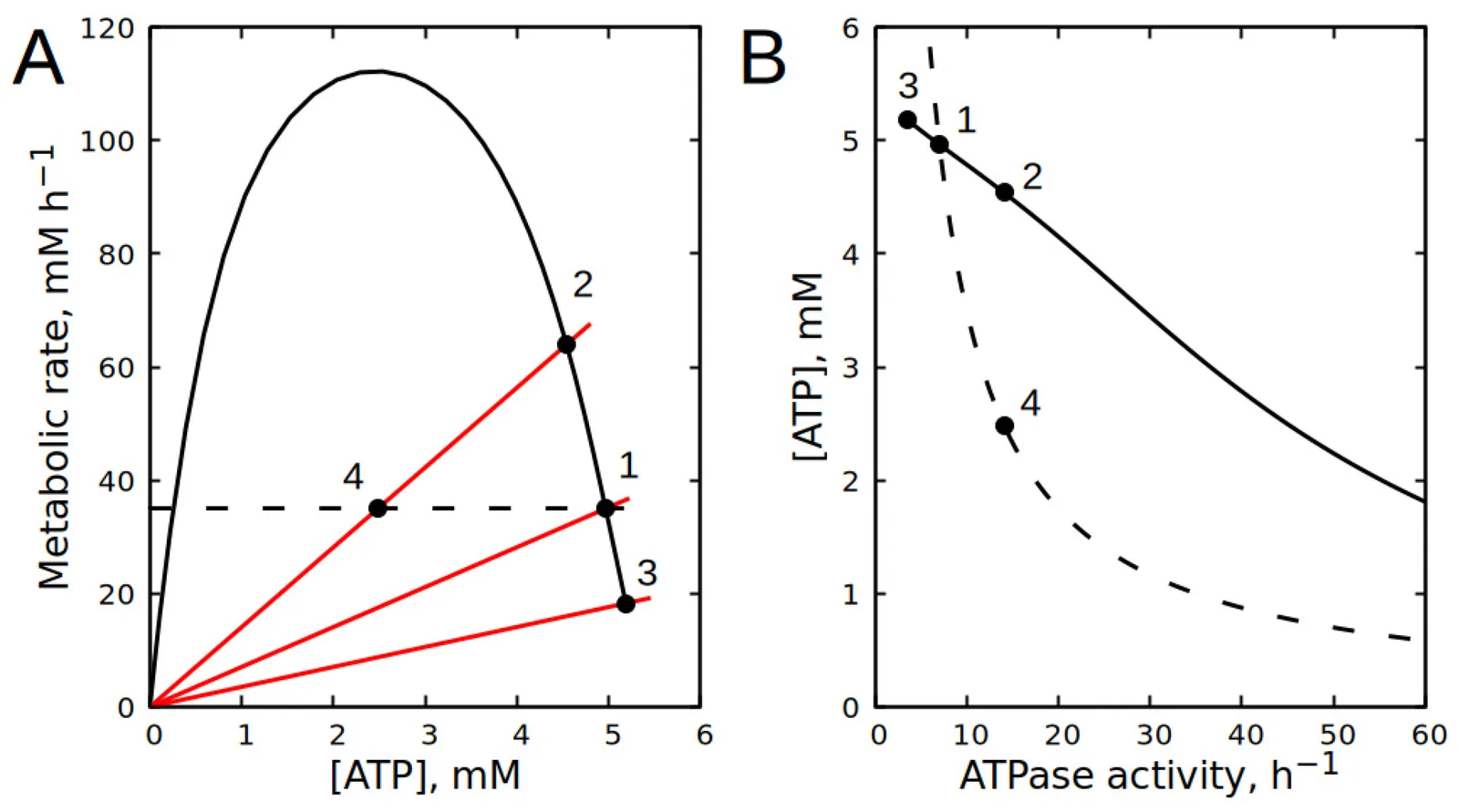
Steady-state characteristics of glycolysis in resting skeletal muscle in the absence of glycogen phosphorylase activity. (**A**) The solid black line shows the dependence of the steady-state ATP production rate in glycolysis on the intracellular ATP concentration (glycolysis characteristic) in the model. The dashed black line indicates ATP production occurring at a constant rate equal to that in resting muscle. Red lines represent ATP consumption rates at resting ATPase activity (1), twofold increased ATPase activity (2), and twofold decreased ATPase activity (3). Intersections of the ATP production and ATP consumption curves define stable steady states of energy metabolism. The corresponding steady-state ATP concentrations are 4.96 mM (1), 4.54 mM (2), 5.19 mM (3), and 2.48 mM (4). The resting steady state (point 1) corresponds to an ATP production/consumption rate of 35 mM·h^−1^ and ATPase activity of 7.05 h^−1^. (**B**) Dependence of the steady-state ATP concentration on ATPase activity. The solid black line shows the dependence calculated using the glycolysis characteristic shown in panel (**A**), whereas the dashed black line represents a hypothetical case with a constant ATP production rate equal to that in resting muscle. Numbered points correspond to the steady states indicated in panel (**A**). Regulation of glycolytic ATP production stabilizes the intracellular ATP concentration over a broad range of ATPase activity. A twofold increase in ATPase activity decreases [ATP] by only ~9% in the regulated system, whereas the same perturbation causes an approximately twofold decrease in [ATP] when ATP production is assumed to be constant.

At zero GP activity, the dependence of the steady-state ATP production rate on [ATP] in the model is described by a bell-shaped curve with a steeply descending branch at physiologically relevant ATP concentrations (Figure 2A). This curve is determined by the rate of ATP production in glycolysis from glucose and is provided due to the inhibition of PFK by ATP and activation by AMP and due to the inhibition of HK by G6P [33,35,36,48]. We refer to this dependence as the glycolysis characteristic because it determines specific features of glycolysis, such as the maximal rate of ATP production and the ability of glycolysis to stabilize [ATP] in cells when the activity of ATP-consuming processes (ATPase) changes [33,35,36,48]. Indeed, the intersection in the graphs of the rates of ATP production and consumption determines the steady-state rate of ATP production and the steady-state ATP concentration in a cell or, in other words, the steady state of energy metabolism in a cell (Figure 2A). Due to the presence of the descending branch on the glycolysis characteristic, the steady-state rate of ATP production in cells is regulated according to the rate of ATP consumption. In Figure 2A, the intersection of the lines corresponding to ATP production and consumption at point 1 determines a steady-state rate of ATP production/consumption equal to 35 mM h^−1^ at an ATP concentration of 4.96 mM. These conditions are considered the resting muscle conditions in the model. The ATPase activity corresponding to these conditions is equal to 7.05 h^−1^. The concentrations of orthophosphate, CrP, ATP, ADP, and AMP in resting skeletal muscle are presented in Table 2. Figure 2 shows that the two-times activation or inhibition of ATPase causes less than a 10% decrease or 5% increase in the ATP level, respectively. Thus, when ATPase activity changes, the ATP concentration in cells is stabilized due to the regulation of the rate of ATP production. The steeper the descending part of the glycolysis characteristic, the better the [ATP] stabilization [35,36]. The height of the “bell” determines the maximal rate of ATP production in glycolysis.

To quantify the ability of the system to stabilize the intracellular ATP concentration in response to changes in ATPase activity, one can use an elasticity coefficient. This coefficient is used in metabolic control analysis to specify how a system variable depends on a specific parameter [49,50,51]. In our case, the elasticity coefficient is described by the following equation:

(1) $$\varepsilon_{ATPase}^{ATP}=\frac{d[ATP]}{\left[ATP\right]}\;/\;\frac{dA_{ATPase}}{A_{ATPase}}\;\;$$

The smaller the absolute value of this coefficient, the better the ATP concentration is stabilized. At the point corresponding to the normal physiological steady state shown in Figure 2, the elasticity coefficient for ATP equals −0.085, meaning that the ATP concentration decreases by 8.5% with a 100% increase in ATPase activity.

Figure 2B shows that, at a constant (unregulated) rate of glycolysis, a change in ATPase activity leads to a proportional change in the cellular ATP concentration—that is, there is no stabilization of the ATP concentration in this case.

### 2.2. Effects of GPa Activation on Steady-State Energy Metabolism in Muscle

**Figure 3:**
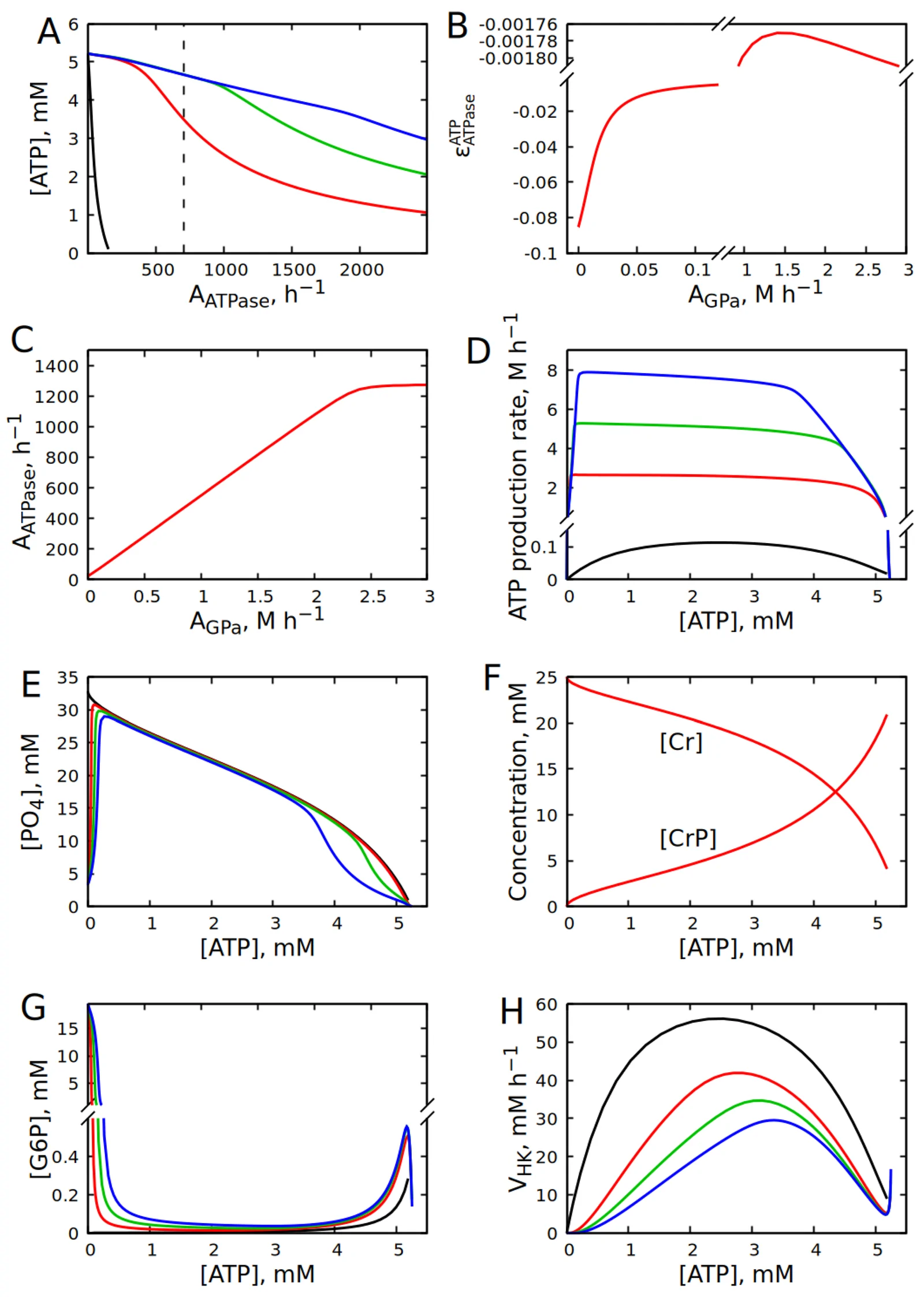
Activation of glycogen phosphorylase a (GPa) enhances ATP homeostasis and alters the steady-state characteristics of skeletal muscle energy metabolism. (**A**) Steady-state ATP concentration ([ATP]) as a function of ATPase activity (A_ATPase_). (**B**) Dependence of the ATP elasticity coefficient with respect to ATPase activity ($\varepsilon_{ATPase}^{ATP}$) on GPa activity (A_GPa_), calculated at the ATPase activity corresponding to resting muscle (7.05 h^−1^). Lower absolute values of the elasticity coefficient indicate the more effective stabilization of the intracellular ATP concentration. (**C**) Dependence of the ATP stabilization range on GPa activity (A_GPa_). The stabilization range is defined as the ATPase activity at which [ATP] decreases by 20% relative to the resting steady-state value. (**D**) Dependence of the steady-state ATP production rate on [ATP]. (**E**–**H**) Dependence of the steady-state concentrations of inorganic phosphate ([PO_4_]) (**E**), creatine and phosphocreatine ([Cr] and [CrP]) (**F**), and glucose 6-phosphate ([G6P]) (**G**) and the steady-state hexokinase reaction rate (V_HK_) (**H**) on [ATP]. Black, red, green, and blue lines represent data obtained at GPa activity of 0, 1.0, 2.0, and 3.0 M·h^−1^, respectively. The vertical dashed line in panel (**A**) indicates ATPase activity that is 100-fold higher than that in resting muscle. In panel (**F**), the curves obtained at different GPa activity levels overlap.

The activation of GPa leads to a pronounced improvement in the stabilization of the steady-state ATP concentration in response to changes in ATPase activity (Figure 3A). At the same time, the activation of GPa substantially expands the range of ATPase activity over which the ATP concentration remains stabilized (Figure 3A). The absolute value of the elasticity coefficient ($\varepsilon_{ATPase}^{ATP}$) decreases markedly with increasing GPa activity up to approximately 0.5 M·h^−1^, beyond which it remains nearly constant (Figure 3B). Thus, the activation of GPa significantly improves ATP stabilization in skeletal muscle. In the considered range of GPa activity from 0 to 3 M h^−1^, the range of ATPase activity in which the stabilization of the ATP concentration is observed increases proportionally to the activity of GPa up to about 2.5 M h^−1^ (Figure 3A,C).

The observed improvement in ATP stabilization is associated with marked changes in the steady-state dependence of the ATP production rate on the intracellular ATP concentration (Figure 3D). First, the activation of GPa results in a substantial increase in the maximal rate of ATP production. Simultaneously, both the steepness and the extent of the descending branch of the dependence of the steady-state ATP production rate on [ATP] increase (Figure 3D). Under conditions of GPa activation, the steady-state dependence of ATP production on [ATP] is largely determined by the corresponding dependence of the steady-state GP reaction rate on [ATP] (Figure S1A).

The stimulation of skeletal muscle contraction can induce up to a 100-fold increase in the ATP consumption rate [23,38,52,53,54]. The model results demonstrate that the activation of GPa can support a corresponding increase in the ATP production rate, which is required to maintain intracellular energy homeostasis during physical activity. Specifically, the ATP consumption rate in resting muscle is approximately 35 mM·h^−1^ [8,24,38,55], and a 100-fold increase corresponds to a rate of 3500 mM·h^−1^. According to the model, this ATP production rate can be achieved at GPa activity of 2000 mM·h^−1^ and higher (Figure 3A,D). Moreover, the activation of GPa enables the stabilization of the intracellular ATP concentration even when ATPase activity is increased by more than 100-fold relative to resting levels (Figure 3A,C,D).

### 2.3. The Important Role of Orthophosphate in Regulating the Rate of Glycogenolysis in Skeletal Muscle

Model analysis reveals an important role of orthophosphate in regulating ATP production and stabilization in muscle cells (Figure 3E). Over a broad range of ATP concentrations, the orthophosphate levels increase as the ATP concentration decreases (Figure 3E). Indeed, the concentration of orthophosphate in muscles increases significantly with a decrease in the concentration of ATP associated with the stimulation of muscle contraction [19,23]. Because orthophosphate serves as a substrate for the GPa and GAPDH reactions and acts as an activator of PFK, an increase in its concentration in the model leads to the acceleration of glycogenolysis via the GPa reaction and to an increase in the rate of ATP production in glycolysis. Consequently, a decrease in the intracellular ATP concentration promotes ATP production from glycogen, which counteracts further ATP depletion and contributes to the stabilization of ATP levels. Thus, orthophosphate participates in the regulation of glycolytic ATP production, in addition to the well-established regulation mediated by ATP and AMP through the PFK reaction [56,57]. In the model, this additional regulatory mechanism results in the enhanced stabilization of the ATP concentration upon activation of the GPa reaction (Figure 3A). At very low ATP concentrations, the model predicts a sharp decrease in orthophosphate levels. This effect is caused by the substantial accumulation of phosphorylated glycolytic intermediates, G6P, G1P, and F6P (Figure 3G and Figure S1B,C). The accumulation of these metabolites ensures the required rate of PFK reaction at low ATP concentrations.

The steady-state concentrations of Cr and CrP closely follow the creatine kinase equilibrium as a function of the intracellular ATP concentration (Figure 3F).

### 2.4. Effects of GPa Activation on HK Reaction Rate and Glucose Consumption

The model predicts that GPa activation leads to an increase in the intracellular concentration of G6P, a glycogen breakdown product (Figure 3G). This increase is observed at all ATP concentrations equal to or below the physiological ATP concentration in resting muscle (Figure 3G). Because G6P inhibits HK, this increase results in a reduction in the HK reaction rate (Figure 3H). Consequently, upon GPa activation, the contribution of blood glucose to ATP synthesis does not change significantly and may even decrease. This model prediction is consistent with experimental observations showing that substantial increases in muscle activity, accompanied by the activation of glycogenolysis, are associated with the accumulation of G6P in skeletal muscle, while the ATP concentration remains almost unchanged or slightly decreased [19,22,38]. At the same time, glucose uptake and utilization remain unchanged or decrease [6,45,46]. From a physiological perspective, this effect implies that the abrupt and intense stimulation of muscle activity does not lead to a pronounced decrease in blood glucose concentrations and therefore does not compromise energy supply to other organs, including the brain.

The steady-state dependencies of additional model variables on the intracellular ATP concentration are presented in the Supplementary Materials (Figure S1D–J).

### 2.5. Effects of GPa Activation on the Kinetics of Muscle Energy Metabolism

**Figure 4:**
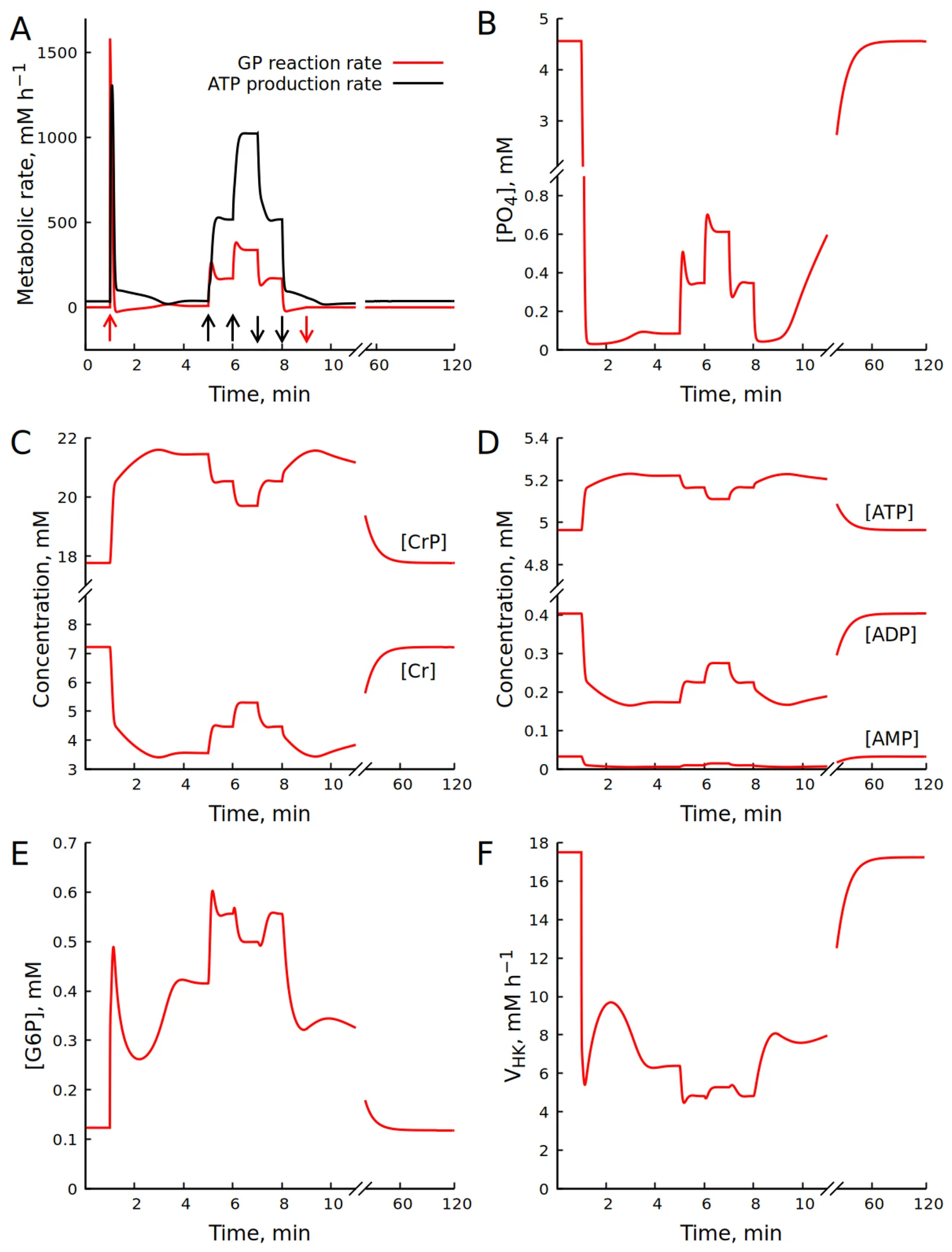
Kinetic responses of muscle energy metabolism to rapid activation and deactivation of GPa and ATPase. (**A**) Rates of the GP reaction and glycolytic ATP production. (**B**–**E**) Time courses of [PO_4_] (**B**); [Cr] and [CrP] (**C**); [ATP], [ADP], and [AMP] (**D**); and [G6P] (**E**). (**F**) Rate of the HK reaction (V_HK_). In panel (**A**), the red arrows indicate the moments of GPa activation and deactivation, whereas the black arrows indicate ATPase activation and deactivation. GPa activity was increased from 0 to 3.0 M h^−1^ and subsequently decreased back to 0. ATPase activity was increased stepwise from 7.05 to 100 and 200 h^−1^ and then decreased to 100 and 7.05 h^−1^.

We next examined how the activation of GPa affects the kinetic behavior of skeletal muscle energy metabolism. Model simulations show that even a substantial increase in GPa activity, in the absence of a concomitant increase in ATP consumption (in resting muscle), induces only a transient increase in the rate of the GP reaction (Figure 4A). Within a few seconds after GPa activation, the GP reaction rate rises sharply and then rapidly declines. The spike in the GP reaction rate coincides with the spike in the rate of ATP production in glycolysis and with a decrease in the orthophosphate concentration (Figure 4A,B). The ATP produced during this spike is primarily utilized for CrP synthesis (Figure 4A,C).

In our model, a sharp decrease in the orthophosphate concentration prevents a significant, sustained increase in the rate of the GP reaction after GPa activation in resting muscle. A significant decrease in the orthophosphate concentration upon GPa activation in resting muscle was also observed experimentally [19,25,58,59]. However, in the experiments, the concentration of orthophosphate decreased much less than in the model. One explanation for these differences may be that the total tissue orthophosphate concentration, including its concentration in both the cytoplasm and the extracellular compartments, was measured in the experiments. In this case, the change in concentration in the cytoplasm may be greater than the total change in concentration in the tissue.

The model predicts that, after GPa activation in resting skeletal muscle, the ATP concentration increases slightly, while the ADP and AMP concentrations decrease markedly (Figure 4D). The transient increase in the GPa reaction rate is accompanied by an increase in G6P levels and a concomitant decrease in the HK reaction rate (Figure 4E,F). In fact, our model predicts a significant increase in the concentrations of all glycolytic intermediates after GPa activation in resting muscle (Figure S2). However, this increase is not large enough to have a significant effect on metabolism or osmotic balance in the muscle. The results of the model analysis are consistent with experimental data demonstrating a significant increase in hexose phosphate levels in resting muscle after adrenaline administration [19,25,58,59].

After GPa deactivation, all metabolite concentrations and reaction rates return to their initial steady-state levels within approximately 50 min (Figure 4).

**Figure 5:**
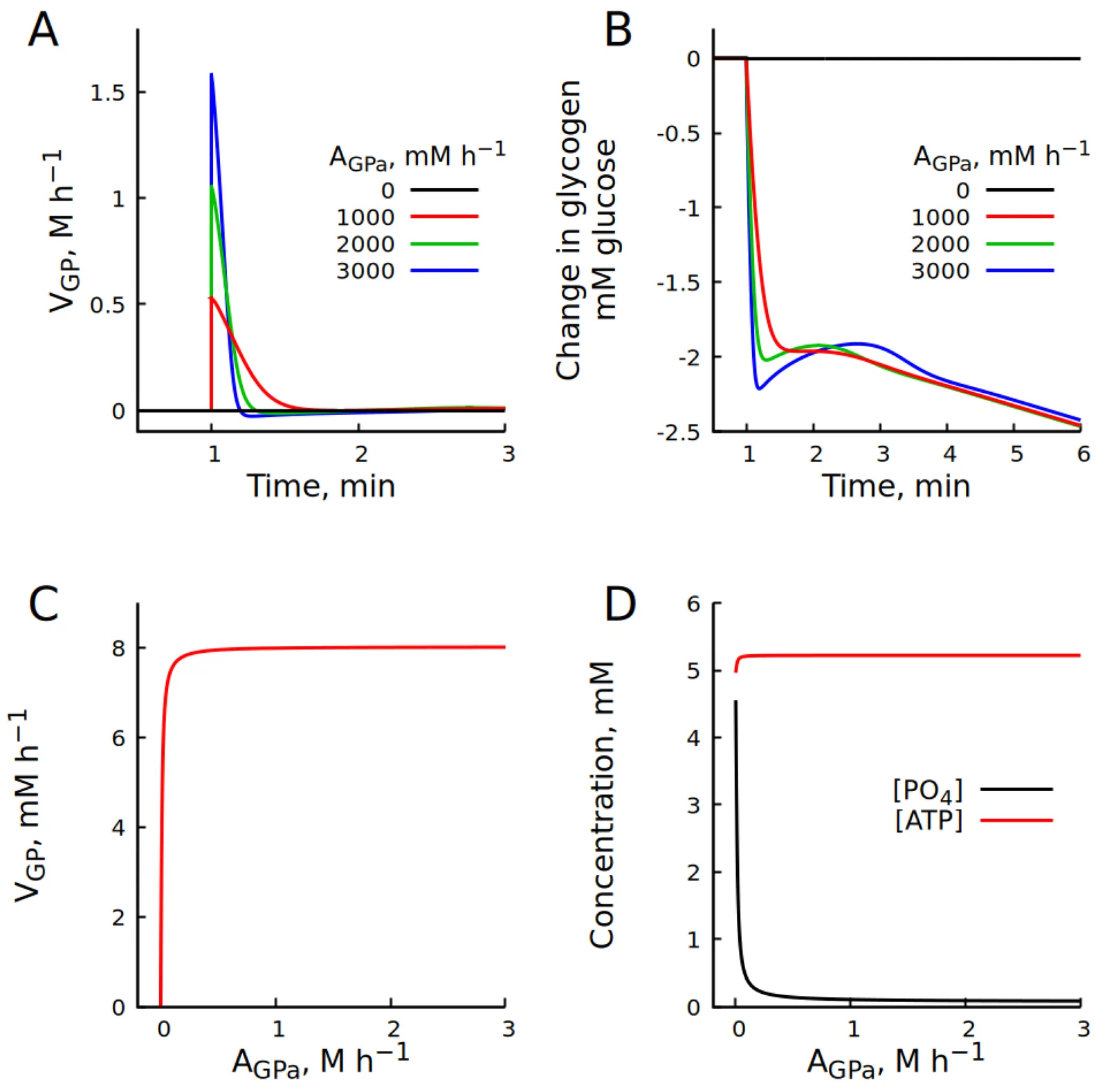
Activation of GPa in resting skeletal muscle induces only a brief transient spike in the GP reaction rate and does not result in significant glycogen consumption. (**A**) Time course of the GP reaction rate following an instantaneous increase in GPa activity (A_GPa_) to different levels in resting skeletal muscle. (**B**) Time course of glycogen consumption (expressed in glucose equivalents) after an instantaneous increase in GPa activity (A_GPa_) in resting skeletal muscle to different levels. (**C**) Dependence of the steady-state GP reaction rate (V_GP_) on GPa activity (A_GPa_) in resting skeletal muscle. (**D**) Dependence of steady-state orthophosphate and ATP concentrations on GPa activity (A_GPa_) in resting skeletal muscle. In panels (**A**,**B**), GPa activity (A_GPa_) was increased at 1 min.

The amplitude of the GP reaction rate spike is proportional to GPa activity (Figure 5A). Notably, after this transient increase, the GP reaction rate may briefly become negative (Figure 4A and Figure 5A), indicating a reversal of the reaction direction toward glycogen synthesis rather than degradation. This decrease, and transient reversal, of the GP reaction rate results from the depletion of the GPa substrate inorganic phosphate, which is consumed during ATP and CrP synthesis (Figure 4B–D). The total amount of glycogen consumed during the transient spike, expressed as glucose equivalents, is approximately 2 mM (Figure 5B), corresponding to less than 5% of the total skeletal muscle glycogen content [21,39,60,61,62,63]. This small net consumption may explain why glycogenolysis has not been experimentally detected following GPa activation in resting skeletal muscle [4,18,19,22,25].

After the transient spike, the GP reaction rate in resting muscle does not fall to zero but stabilizes at relatively low values that depend on GPa activity (Figure 5B,C). This behavior arises because GPa activation increases the G6P concentration (Figure 3G and Figure 4E), which inhibits HK activity (Figure 3H and Figure 4F). As a consequence, ATP production from blood glucose becomes insufficient to maintain energy balance, and a compensatory contribution from glycogen-derived ATP production is required. At GPa activity exceeding ~500 mM h^−1^, the steady-state GP reaction rate in resting muscle becomes nearly independent of GPa activity and stabilizes at approximately 8 mM h^−1^ (Figure 5C). Under these conditions, a new steady state is established, characterized by markedly reduced inorganic phosphate levels and a slight increase in ATP concentrations (Figure 5D). Importantly, even this maximal steady-state GP reaction rate is comparable to reported rates of glycogen synthesis in skeletal muscle [4,61,64,65] and is therefore unlikely to result in net glycogen depletion.

In contrast, increasing ATP consumption in the presence of activated GPa produces a sustained elevation in both the GP reaction rate and ATP production rate (Figure 4A). The activation of ATP-consuming processes leads to a decrease in CrP levels and a concomitant increase in the inorganic phosphate concentration (Figure 4B,C). The resulting elevation in phosphate availability supports sustained GPa activity and stable glycogen consumption. Under these conditions, the steady-state GP reaction and ATP production rates are proportional to the rate of ATP consumption (Figure 4A). Experimental data available in the literature show an increase in the level of orthophosphate and a decrease in the level of phosphocreatine during the stimulation of muscle contraction [19,22,23,38,58,59,66]. Thus, our model results are in qualitative agreement with experimental data.

The deactivation of ATPase leads to a corresponding decline in both the GP reaction and ATP production rates (Figure 4A).

The activation and deactivation of GPa, as well as the modulation of ATP-consuming processes in the presence of activated GPa, induce only minor changes in the ATP concentration (Figure 4D). Throughout these transitions, the cellular energy charge remains tightly regulated, varying within a narrow range from 0.957 to 0.984. Thus, the regulatory architecture of muscle energy metabolism ensures the stabilization of the ATP levels and energy charge both during transient processes and at steady state. These model results are also in good agreement with the experimental data available in the literature [19,22,23,24,38,59,66,67,68].

It should be noted that, due to the short characteristic times (1–60 s), muscle energy metabolism functions essentially in a steady state. When ATP consumption is activated or deactivated, new steady states are established within tens of seconds (Figure 4). As a result, the equality between the rates of production and consumption of ATP is achieved in approximately 10 s (Figure S3A). Figure S3B shows that the practically steady-state dependence of the ATP concentration on ATPase activity (analogous to Figure 3A) is achieved less than 20 s after a simultaneous abrupt increase in GPa and ATPase activity.

### 2.6. Effects of Elevated Inorganic Phosphate Levels on the Rate of Glycogenolysis in Skeletal Muscle

**Figure 6:**
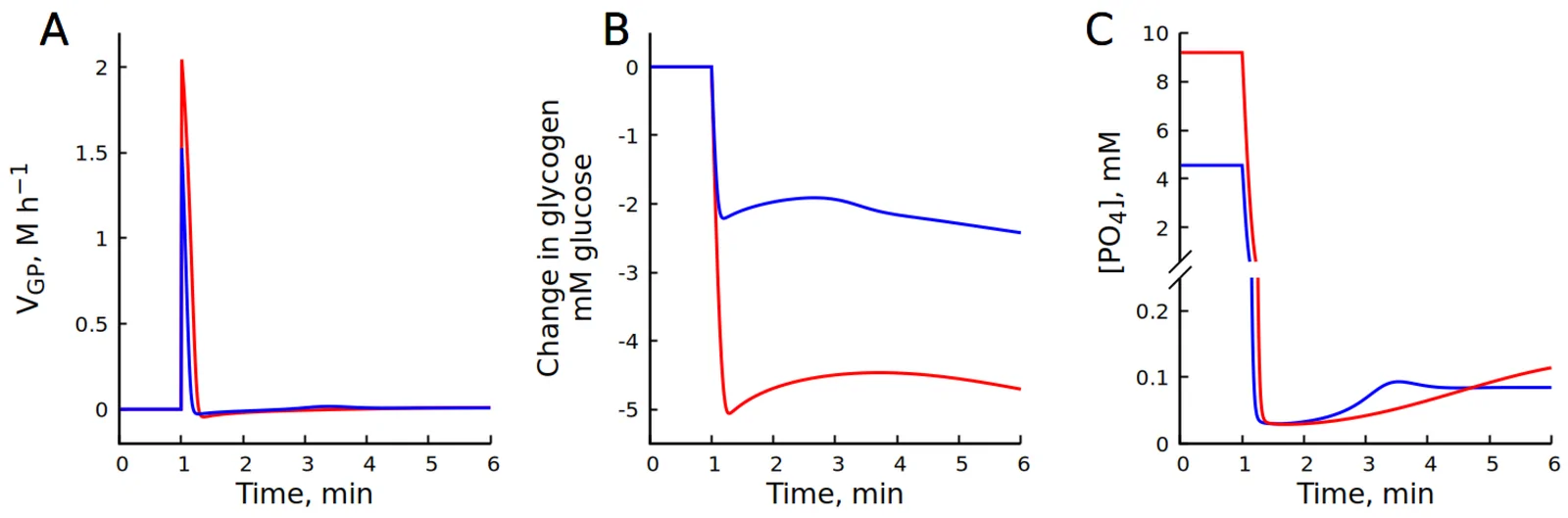
An increase in orthophosphate concentration in the model does not induce substantial glycogen consumption under resting conditions. (**A**–**C**) Time courses of the GP reaction rate (**A**), glycogen consumption (**B**), and orthophosphate concentration (**C**) following an instantaneous increase in GPa activity from 0 to 3.0 M h^−1^ at 1 min. The blue and red lines correspond to simulations performed at normal (4.56 mM) and elevated (9.18 mM) orthophosphate concentrations in resting skeletal muscle, respectively. The total intracellular phosphate pool (parameter Q (Equation (2), Table 3)) was increased from 32.9 to 37.7 mM in simulations with elevated orthophosphate concentrations.

We next assessed the effects of elevated concentrations of inorganic phosphate, a substrate of the GP reaction, on glycogen consumption in the model. It was shown earlier that circulatory occlusion in muscles causes about a two-fold increase in orthophosphate levels [19]. However, the subsequent activation of GPa with adrenaline infusion did not stimulate pronounced activation of glycogen consumption without the stimulation of muscle contraction [19]. Our model explains these results as follows. In the absence of GPa activation, increasing phosphate concentrations have no effect on glycogen consumption, because the non-phosphorylated GPb form is inactive in the model. Following GPa activation, a higher phosphate concentration amplifies the GP reaction rate spike (Figure 6A) and significantly increases the amount of glycogen consumed during this transient phase (Figure 6B). However, even with a doubled initial phosphate concentration, after the activation of GPa, it drops to levels that can provide only a very low rate of GP reaction (Figure 6C). Glycogen consumption during the spike in the GP reaction rate remains modest, accounting for only ~5–10% of the total muscle glycogen content. The steady-state glycogen consumption rate established after the spike increases to approximately 9.8 mM h^−1^, compared with 8.0 mM h^−1^ at baseline phosphate levels. This difference reflects the higher steady-state phosphate concentrations achieved after GPa activation (Figure 6C). Nevertheless, both rates remain close to the reported glycogen synthesis rates in muscle [4,61,64,65] and are therefore unlikely to cause net glycogen depletion.

It should be noted that, at elevated orthophosphate levels, our model predicts a strong decrease in orthophosphate concentrations upon the activation of GPa by adrenaline in resting muscle (Figure 6C). At the same time, experiments show a relatively small decrease in orthophosphate levels [19]. As noted above, we believe that this discrepancy can be explained by the fact that the model predicts the concentration of orthophosphate in the cytoplasm, while experiments measure the total concentration of orthophosphate in muscle tissue, including the concentrations of orthophosphate in the cytoplasm and in the extracellular compartment.

### 2.7. Contributions of CK and AK Reactions to ATP Production in Skeletal Muscle After Stimulation of Contraction

**Figure 7:**
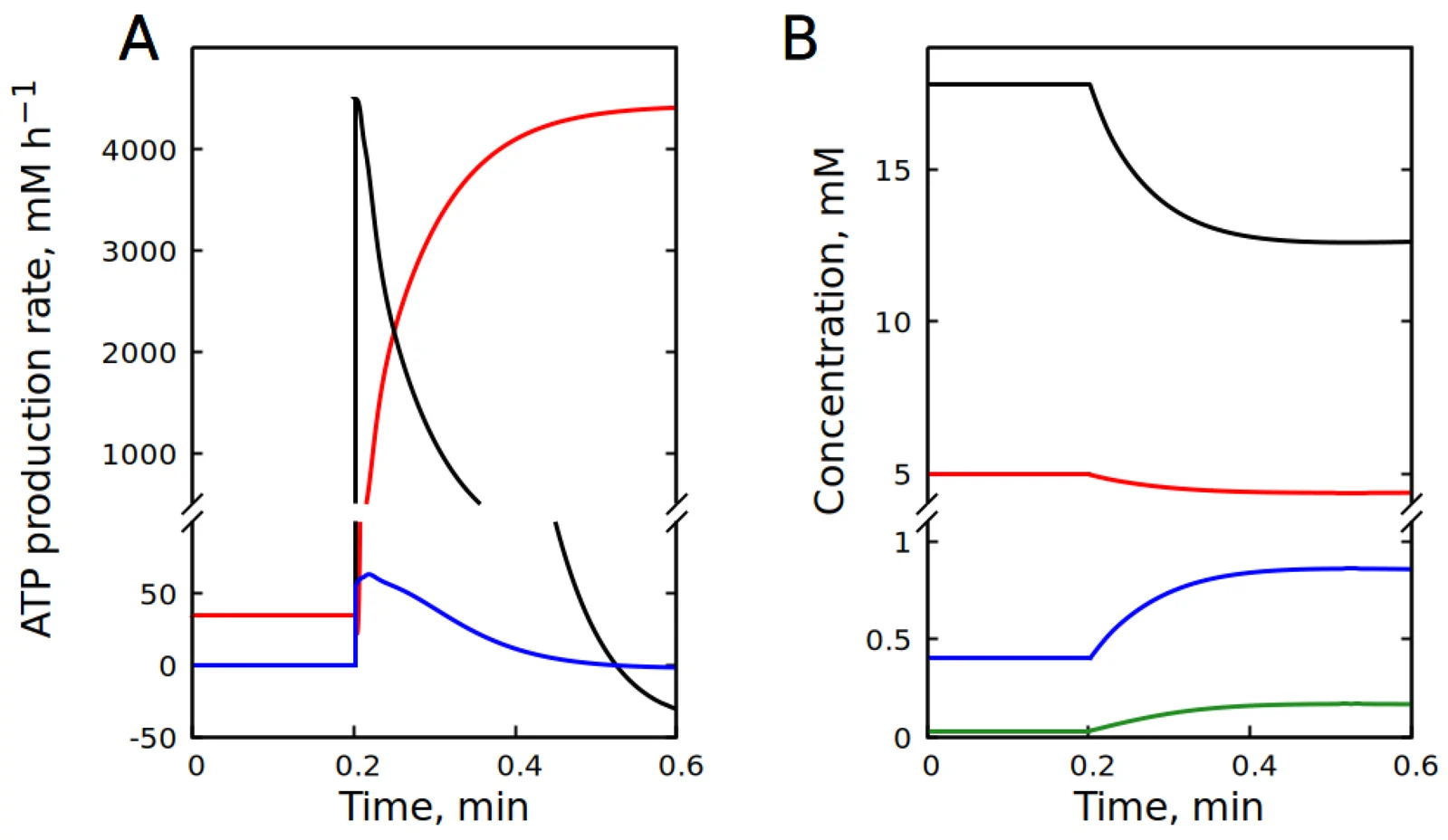
Contributions of CK, AK, and glycolysis to ATP production following simultaneous activation of ATPase and GPa. (**A**) Rates of ATP production via the CK reaction (black line), AK reaction (blue line), and glycolysis (red line). (**B**) Time courses of [CrP] (black line), [ATP] (red line), [ADP] (blue line), and [AMP] (green line) after a simultaneous instantaneous increase in A_GPa_ from 0 to 3.0 M h^−1^ and A_ATPase_ from 7.05 to 1000 h^−1^ at time equal to 0.2 min.

The simultaneous activation of ATP-consuming processes and GPa highlights the primary role of phosphocreatine as an immediate energy source during abrupt increases in muscle activity (Figure 7). Under these conditions, ATP production initially rises sharply due to CrP breakdown and is subsequently replaced by ATP production via glycolysis within approximately 10 s (Figure 7A), consistent with experimental observations [7,21,54]. In these simulations, ATPase activity was increased ~140-fold relative to resting levels. The CrP concentration decreased by ~30%, whereas the ATP concentration declined by only ~12% (Figure 7B), and the energy charge decreased from 0.957 to 0.890 (~7%). Although the AK reaction has been proposed as an auxiliary ATP source during contraction onset [7,54,69], our results indicate that its contribution is negligible compared with those of CK and glycolysis (Figure 7A).

### 2.8. Effects of Forced Acceleration of the GP Reaction on Energy Metabolism in Resting Muscle

**Figure 8:**
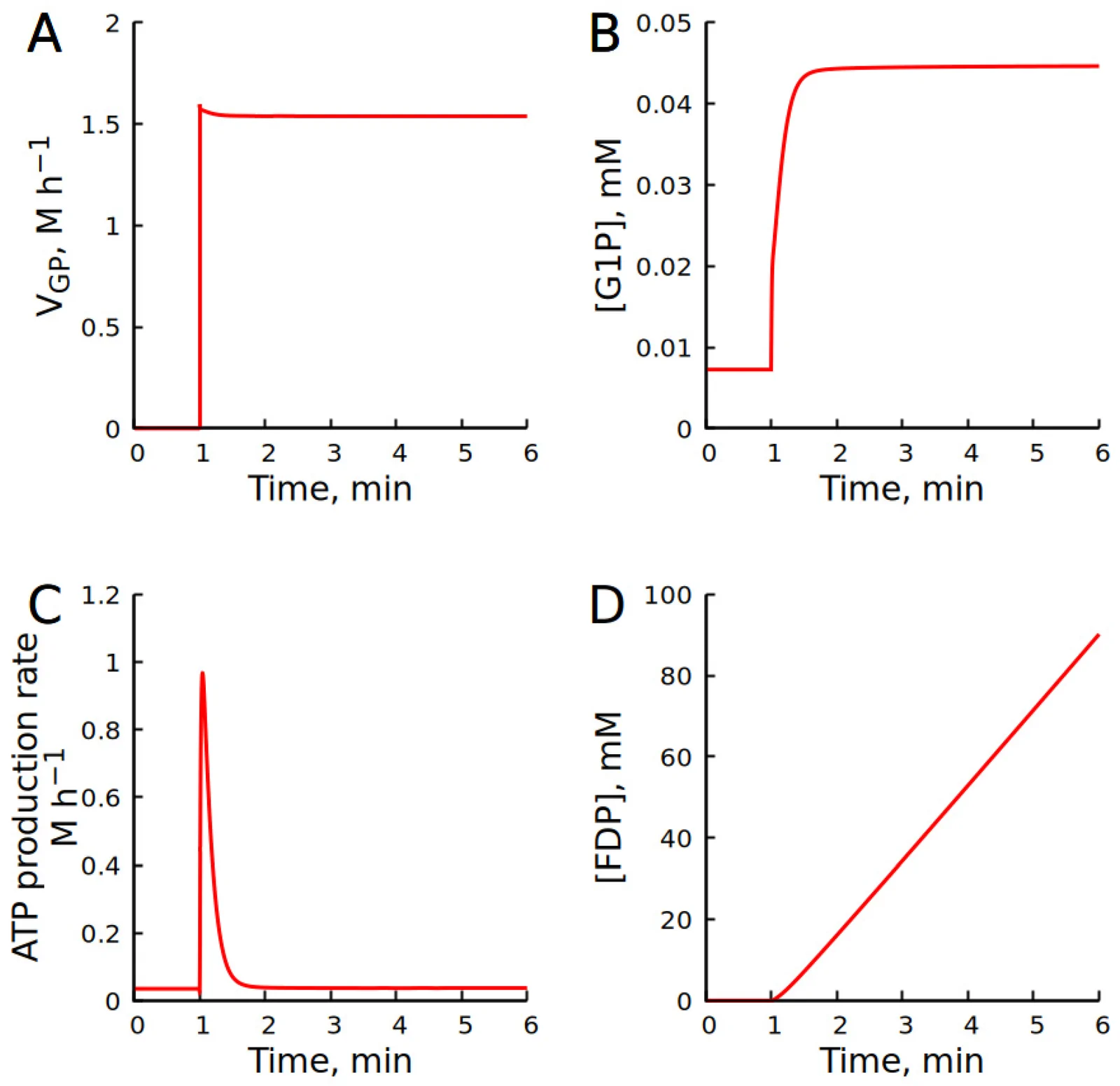
Activation of GPa in resting muscle at a constant orthophosphate concentration leads to a prolonged increase in the GP reaction rate and the accumulation of metabolites that may cause glycogen depletion, osmotic swelling, and subsequent muscle cell damage. (**A**–**D**) Time courses of the GP reaction rate (V_GP_) (**A**), G1P concentration (**B**), rate of ATP production (**C**), and FDP concentration (**D**) in the model under rest conditions at constant [PO_4_] = 4.56 mM following an instantaneous increase in GPa activity to 3.0 M h^−1^ at 1 min. Under these conditions, the total phosphate pool (parameter Q (Equation (2), Table 3)) was not constrained to remain constant.

Finally, the model predicts that the forced acceleration of the GP reaction in resting muscle may be deleterious. To ensure the sustained functioning of the GP reaction in resting muscle, we set the orthophosphate concentration constant at 4.56 mM in the model. Assuming unlimited phosphate availability (constant phosphate concentration), strong GPa activation (up to 3.0 M h^−1^) produces a prolonged increase in the GP reaction rate, leading to the substantial accumulation of metabolic intermediates, particularly FDP (Figure 8 and Figure S4A–C). After an initial rise, the GP reaction rate decreases slightly (Figure 8A) due to the accumulation of G1P (Figure 8B), which inhibits glycogen breakdown. The increase in the GP reaction rate generates a brief spike in the ATP production rate (Figure 8C) and the sustained accumulation of FDP, DAP, GAP, and 1,3DPG (Figure 8D and Figure S4A). The concentrations of other glycolytic intermediates increase transiently and then stabilize (Figure S4B,C).

During the spike, the ATP production rate reaches ~1000 mM h^−1^ but rapidly returns to near-resting levels within ~1 min (Figure 8C). The ATP produced during the spike causes an increase in [CrP] (Figure S4D). The accumulation of 1,3DPG, DAP, GAP, and FDP reflects the activation of PFK driven by increased flux through the GP reaction and elevated F6P levels, whereas flux through the lower part of glycolysis remains constrained due to low ADP levels (Figure S4E–G). Despite stable ATP levels, the sustained accumulation of osmotically active metabolites occurs. Notably, the FDP concentration reaches ~100 mM within minutes (Figure 8D), approaching the maximal skeletal muscle glycogen content expressed in glucose equivalents [21,39,60,61,62,63]. This means that all muscle glycogen is depleted to zero due to futile conversion to FDP. Moreover, such accumulation of an osmotically active metabolite may cause the destruction of muscle cells due to osmotic swelling.

Thus, a prolonged increase in the rate of GP reaction without activation of the ATP consumption rate in muscles is expected to have catastrophic consequences for cells. The regulation of muscle energy metabolism appears to protect muscle cells from such situations.

In summary, our results demonstrate that increased GPa activity alone, without the concurrent activation of ATP-consuming processes, does not induce sustained increases in the GP reaction rate, glycogenolysis, or ATP production. It should be noted that the activation of GP without the concomitant activation of ATP-consuming processes is quite realistic, since inactive GPb can be converted into highly active GPa due to an increase in blood adrenaline levels under stress conditions. In such situations, the activation of GP may be considered a switch in metabolism to a metabolic standby mode and preparation for intense muscle activity. However, the activation of glycogenolysis and ATP production must be prevented in this mode based on our model.

### 2.9. Sensitivity of Model Results to Variations in Model Parameter Values

An important issue for any model is the sensitivity of the modeling results to changes in the values of the model parameters. From Table 3, it can be seen that, for most parameters of our model, the range of experimental values is quite large. However, variations in the equilibrium constants of reversible enzymatic reactions cannot have a significant impact on the qualitative behavior of the model. They will only lead to changes in the ratios between the concentrations of the corresponding metabolites. Therefore, we investigated the dependence of the modeling results on enzyme activity. To this end, we tested the model with the set of minimum and maximum enzyme activity values presented in Table 3.

Our study showed that the modeling results are qualitatively independent of the enzyme activities in the model (Figure S5). The shape of the steady-state dependences of the ATP production rate on the ATP concentration and the ATP concentration on ATPase activity is qualitatively preserved for different sets of parameters (Figure S5A,B). The kinetic behavior of the model is also preserved (Figure S5C–F). In all cases, the activation of GPa by itself does not lead to a long-term increase in the rates of glycogenolysis and ATP production (Figure S5C,D). This is associated with a decrease in the concentration of orthophosphate in the cytoplasm (Figure S5E). A steady-state increase in the rates of glycogenolysis and ATP production in all cases occurs only with an increase in the concentration of orthophosphate in the cytoplasm due to the activation of ATP consumption (Figure S5C–E). The lower the GPa activity, the greater the increase in phosphate concentration required to ensure the same rate of the GP reaction (Figure S5E). It should be noted, however, that, with a minimum set of parameters, the stabilization of the ATP level in the model significantly worsens (Figure S5F). Thus, we can state that the main conclusions of the model do not change when changing the model parameter values within the experimental range.

## 3. Discussion

Our results identify ATP consumption as the key driver of glycogenolysis in skeletal muscle. The activation of glycogen phosphorylase alone is not sufficient. Sustained glycogen breakdown requires a concomitant increase in ATP demand.

In resting muscle, glycolysis from blood glucose can maintain ATP levels over a limited range of ATPase activity. However, this capacity is restricted to relatively small increases in energy demand. In contrast, intense muscle contraction may require a 50–100-fold increase in ATP turnover. This demand cannot be met by glycolysis without the activation of glycogenolysis. The activation of GPa expands both the maximal rate of ATP production and the range of ATPase activity over which ATP levels remain stabilized.

A central finding of this study is the role of inorganic phosphate as a metabolic regulator linking ATP consumption to glycogenolysis. In contracting muscle, ATP hydrolysis increases intracellular phosphate levels. This directly stimulates the GP reaction and thereby enables sustained glycogen breakdown. At the same time, phosphate activates key glycolytic steps, further enhancing ATP production. As a result, the ATP supply is automatically matched to the demand.

In resting muscle, this coupling is absent. Phosphate levels remain low and, according to the model, are rapidly depleted upon GPa activation. As a consequence, glycogenolysis cannot be sustained, even when GPa is fully active. This mechanism allows us to explain why neither increased phosphate, AMP, nor GPa activation alone induces glycogen breakdown in resting muscle, and it eliminates the need to invoke unknown contraction-specific factors [4,18]. However, to confirm this mechanism, information about the kinetics of the intracellular orthophosphate concentration in contracting muscle cells is required. In the absence of such information, we cannot exclude the existence of additional contraction-specific factors regulating glycogen consumption.

The model also predicts that uncoupling glycogenolysis from the ATP demand is potentially harmful. Forced activation of the GP reaction under conditions of limited ATP turnover leads to the accumulation of phosphorylated glycolytic intermediates. This accumulation arises from continued flux through the upper part of glycolysis in the presence of limited ADP availability downstream. The resulting buildup of osmotically active metabolites may lead to cellular swelling and damage. Thus, the requirement for ATP consumption acts as a protective constraint on glycogen utilization.

These results provide a mechanistic explanation for the well-known observation that the adrenaline-induced activation of glycogen phosphorylase in resting muscle does not lead to glycogenolysis [4,18,19,22,25]. We propose that this state represents a metabolic standby mode. In this mode, the enzymatic machinery for glycogen breakdown is activated, but substrate availability (phosphate) remains limiting. This prevents premature glycogen consumption while enabling a rapid response to subsequent increases in ATP demand.

Our analysis further reveals a functional interaction between glycogenolysis and glucose uptake. The activation of GPa increases intracellular G6P levels, thereby inhibiting hexokinase. This limits glucose utilization from the blood during intense muscle activity. Such regulation likely helps to preserve systemic glucose homeostasis and ensures adequate glucose supply to other organs, particularly the brain.

The model also captures the metabolic role of creatine phosphate during rapid transitions from rest to intense contraction. The creatine kinase reaction provides an immediate buffer for ATP during sudden increases in demand, whereas glycolysis becomes dominant within seconds. In contrast, the contribution of adenylate kinase to ATP production is negligible under these conditions.

More broadly, the model highlights a general principle of metabolic regulation in muscle. ATP production and consumption are tightly coupled through shared metabolites. Among these, inorganic phosphate emerges as a key coordinator of the energy state and metabolic flux. Through this coupling, ATP consumption effectively sets the rate of ATP production.

Finally, we conclude that a relatively simple qualitative model that incorporates key enzymatic properties and metabolite constraints can account for a wide range of experimental observations. The model explains both the steady-state and transient behaviors of muscle energy metabolism and provides a unified framework for understanding the regulation of glycogenolysis during muscle activity.

## 4. Materials and Methods

### 4.1. General Features

The mathematical model describes a simplified scheme of the energy metabolism in mammalian white skeletal muscle (Figure 1). This means that we do not take into account the possible contribution of oxidative phosphorylation to ATP production. In addition, we do not consider reactions of glycogen synthesis, because we aim to investigate the influence of glycogen degradation on skeletal muscle energy metabolism under the stimulation of muscle activity.

The model includes the first two enzymatic reactions of glycolysis, catalyzed by hexokinase (HK) and phosphofructokinase (PFK), which determine the glycolytic flux [33,48], glyceraldehyde 3-phosphate dehydrogenase (GAPDH); two ATP-generating reactions, namely the phosphoglycerate kinase (PGK) and pyruvate kinase (PK), glycogen phosphorylase (GP) reaction and the sum of the ATP-consuming processes, indicated as ATPase. The fast reversible enzymatic reactions catalyzed by glucose phosphate isomerase (GPI) and phosphoglucomutase (PGLM), aldolase (ALD) and triosephosphate isomerase (TPI), phosphoglycerate mutase (PGM), and enolase (ENO) are replaced by the corresponding equilibrium relationships between metabolite concentrations. We consider the pyruvate kinase (PK) reaction to be irreversible and omit lactate dehydrogenase. We assume a linear dependence of the ATPase reaction rate on [ATP].

Moreover, the model includes adenylate kinase (AK) and creatine kinase (CK) equilibria. The sums of the concentrations [ATP] + [ADP] + [AMP] and [Cr] + [CrP] are assumed to be constant. Indeed, a number of studies have shown that, when stimulating muscle contraction (except for extreme loads), these sums do not change within the experimental error [23,24,38,66,67,68]. The concentrations of glucose, glycogen, NAD, NADH, and H^+^ are also assumed to be constant. The list of the main model parameters and their values is presented in Table 3.

We neglect the possible effects of metabolites other than those in the model and of inorganic ions (except orthophosphate) on the reaction rates in the model.

### 4.2. Balance of Phosphate in the Model

Orthophosphate enters muscle cells via co-transport with Na^+^ ions, provided by two transporters, Pit1 and Pit2 [70]. The maximal rate of orthophosphate transport into skeletal muscle cells is about 2 mmol h^−1^ kg^−1^ cells [71]. Our estimates of the rate of ATP turnover in resting muscle based on the rate of glycolysis [8,24,38,55] give a value in the range of 8 to 58 mmol h^−1^ kg^−1^ tissue. In contracting skeletal muscle, the rate of ATP turnover can increase by up to 10^4^ mmol·h^−1^·kg^−1^ of tissue [22,23,38,39]. Therefore, we consider the rate of orthophosphate transport in skeletal muscle to be negligible compared to the phosphate turnover rate in energy metabolism, and we assume that the total amount of phosphate in metabolites involved in energy metabolism in the cell is constant:

(2) $$Q=const=\left[PO_{4}\right]+\left[G1P\right]+\left[G6P\right]+\left[F6P\right]+2\left[FDP\right]+\left[DAP\right]\;+\;\left[GAP\right]+2\left[1,3DPG\right]+\left[3PG\right]+\left[2PG\right]+\left[PEP\right]+2\left[ATP\right]+\left[ADP\right]+\left[CrP\right]$$

In ATP and ADP molecules, we take into account only labile phosphate that can be involved in energy metabolism.

### 4.3. Base Equations

The model is based on the following system of equations for the concentrations of the metabolites shown in Figure 1:

(3) $$\frac{d\left[G1P\right]}{dt}=V_{GPa}-V_{PGLM}$$

(4) $$\frac{d\left[G6P\right]}{dt}=V_{HK}+V_{PGLM}-V_{GPI}$$

(5) $$\frac{d\left[F6P\right]}{dt}=V_{GPI}-V_{PFK}$$

(6) $$\frac{d\left[FDP\right]}{dt}=V_{PFK}-V_{ALD}$$

(7) $$\frac{d\left[DAP\right]}{dt}=V_{ALD}-V_{TPI}$$

(8) $$\frac{d\left[GAP\right]}{dt}=V_{ALD}+V_{TPI}-V_{GAPDH}$$

(9) $$\frac{d\left[1,3DPG\right]}{dt}=V_{GAPDH}-V_{PGK}$$

(10) $$\frac{d\left[2PG\right]}{dt}=V_{PGK}-V_{PGM}$$

(11) $$\frac{d\left[3PG\right]}{dt}=V_{PGM}-V_{ENO}$$

(12) $$\frac{d\left[PEP\right]}{dt}=V_{ENO}-V_{PK}$$

(13) $$\frac{d\left[ATP\right]}{dt}=-V_{HK}-V_{PFK}+V_{PGK}+V_{PK}-V_{ATPase}+V_{AK}+V_{CK}$$

(14) $$\frac{d\left[ADP\right]}{dt}=V_{HK}+V_{PFK}-V_{PGK}-V_{PK}+V_{ATPase}-2V_{AK}-V_{CK}$$

(15) $$\frac{d\left[AMP\right]}{dt}=V_{AK}$$

(16) $$\frac{d\left[Cr\right]}{dt}=V_{CK}$$

(17) $$\frac{d\left[CrP\right]}{dt}=-V_{CK}$$

(18) $$\left[Glycogen\right]=const$$

(19) $$\left[Glucose\right]=const$$

(20) $$\left[NAD\right]=const$$

(21) $$\left[NADH\right]=const$$

Here, *V_X_* denotes the rate of reaction catalyzed by enzyme X.

### 4.4. Reduction of the Mathematical Model

Taking into account that many enzymatic reactions in the model are considered to be at equilibrium, a number of simplifications can be made in the equations.

1. As mentioned above, we assume that the activity of PGLM and GPI is high enough to provide the equilibrium ratios between the substrates and products of these reactions. To exclude the PGLM and GPI reaction rates from the model equations, we sum Equations (3)–(5). This results in the following equation:

(22) $$\frac{du}{dt}=V_{HK}+V_{GP}-V_{PFK}$$

where

(23) $$u=\left[G6P\right]+\left[F6P\right]+\left[G1P\right]$$

The concentrations of G6P, F6P, and G1P are calculated using Equation (23) and the following equilibrium relationships for the PGLM and GPI reactions:

(24) $$K_{PGLM}=\frac{\left[G6P\right]}{\left[G1P\right]}\;$$

(25) $$K_{GPI}=\frac{\left[F6P\right]}{\left[G6P\right]}$$

As a result, we obtain the following mathematical expressions:

(26) $$\left[G1P\right]=u\frac{1}{1+K_{PGLM}\left(1+K_{GPI}\right)}$$

(27) $$\left[G6P\right]=u\frac{K_{PGM}}{1+K_{PGLM}\left(1+K_{GPI}\right)}\;$$

(28) $$\left[F6P\right]=u\frac{K_{PGLM}{\;K}_{PGI}}{1+K_{PGLM}\left(1+K_{GPI}\right)}$$

2. We also assume that the ALD and TPI reactions are in equilibrium. To exclude the ALD and TPI reaction rates from the model equations, we multiply Equation (6) by two and sum it with Equations (7) and (8). This results in the following equation:

(29) $$\frac{dw}{dt}=2V_{PFK}-V_{GAPD}$$

where

(30) $$w=2\left[FDP\right]+\left[DAP\right]+\left[GAP\right]$$

The concentrations of GAP, DAP, and FDP are calculated using Equation (30) and the following equilibrium relationships for the ALD and TPI reactions:

(31) $$K_{ALD}=\frac{\left[GAP\right]\left[DAP\right]}{\left[FDP\right]}$$

(32) $$K_{TPI}=\frac{\left[GAP\right]}{\left[DAP\right]}$$

As a result, we obtain the following mathematical expressions:

(33) $$\left[GAP\right]=\frac{1}{4}\left(B-K_{ALD}\left(1+K_{TPI}\right)\right)$$

(34) $$\left[DAP\right]=\frac{1}{4K_{TPI}}\left(B-K_{ALD}\left(1+K_{TPI}\right)\right)$$

(35) $$\left[FDP\right]=\frac{1}{8K_{TPI}}\left(K_{ALD}{\left(1+K_{TPI}\right)}^{2}+4K_{TPI}w-(1+K_{TPI}\right)B)\;$$

where

(36) $$B=\sqrt{K_{ALD}\left(K_{ALD}{\left(1+K_{TPI}\right)}^{2}+8K_{TPI}w\right)}$$

3. Next, we assume that the PGM and ENO reactions are in equilibrium. To exclude the PGM and ENO reaction rates from the model equations, we sum Equations (10)–(12). This results in the following equation:

(37) $$\frac{dq}{dt}=V_{PGK}-V_{PK}$$

where

(38) $$q=\left[3PG\right]+\left[2PG\right]+\left[PEP\right]$$

The concentrations of 3PG, 2PG, and PEP are calculated using Equation (38) and the following equilibrium relationships for the PGM and ENO reactions:

(39) $$K_{PGM}=\frac{\left[2PG\right]}{\left[3PG\right]}$$

(40) $$K_{ENO}=\frac{\left[PEP\right]}{\left[2PG\right]}$$

As a result, we obtain the following mathematical expressions:

(41) $$\left[3PG\right]=q\frac{1}{1+K_{PGM}\left(1+K_{ENO}\right)}$$

(42) $$\left[2PG\right]=q\frac{K_{PGM}}{1+K_{PGM}\left(1+K_{ENO}\right)}$$

(43) $$\left[PEP\right]=q\frac{K_{PGM}K_{ENO}}{1+K_{PGM}\left(1+K_{ENO}\right)}$$

4. Finally, we assume that the AK and CK reactions are in equilibrium. To exclude the AK and CK reaction rates from the model equations, we multiply Equation (13) by two and sum it with Equations (14) and (17). This results in the following equation:

(44) $$\frac{de}{dt}=-V_{HK}+3V_{PFK}-V_{ATPase}$$

where

(45) $$e=2\left[ATP\right]+\left[ADP\right]+\left[CrP\right]$$

The concentrations of ATP, ADP, AMP, Cr, and CrP can be calculated using Equation (45), the following equilibrium relationships for the PGM and ENO reactions, and the following two conservation equations for metabolites involved in energy turnover:

(46) $$K_{AK}=\frac{\left[ATP\right]\left[AMP\right]}{{\left[ADP\right]}^{2}}$$

(47) $$K_{CK}\left[H^{+}\right]=K_{CK}^{\prime }=\frac{\left[Cr\right]\left[ATP\right]}{\left[CrP\right]\left[ADP\right]}$$

(48) $$\left[ATP\right]+\left[ADP\right]+\left[AMP\right]=P=const$$

(49) $$\left[Cr\right]+\left[CrP\right]=C=const$$

In our calculations, we assume that [H^+^] is constant.

Using Equations (46)–(49), one can express concentrations of ATP, ADP, AMP, and Cr through the concentration of CrP as follows:

(50) $$\left[Cr\right]=\;C-\left[CrP\right]$$

(51) $$\left[ATP\right]=\frac{{\left[CrP\right]}^{2}K_{CK}^{\prime 2}P}{D}\;$$

(52) $$\left[ADP\right]=\frac{\left(C-\left[CrP\right]\right)\left[CrP\right]K_{CK}^{\prime }P}{D}\;\;$$

(53) $$\left[AMP\right]=\frac{{\left(C-\left[CrP\right]\right)}^{2}K_{AK}P}{D}$$

where

(54) $$D=C^{2}K_{AK}+C\left[CrP\right]\left(K_{CK}^{\prime }-2K_{AK}\right)+{\left[CrP\right]}^{2}\left(K_{AK}+\left(K_{CK}^{\prime }-1\right)K_{CK}^{\prime }\right)$$

By substituting Equations (51) and (52) for [ATP] and [ADP] into (45) and rearranging the resulting equation, one can obtain the cubic equation regarding [CrP], which can be solved to obtain the concentration of CrP as a function of the variable *e*:

(55) $$a{\left[CrP\right]}^{3}+b{\left[CrP\right]}^{2}+c\left[CrP\right]+d=0$$

where

(56) $$a=\left(1-K_{CK}^{\prime }\right)K_{KC}^{\prime }-K_{AK}$$

(57) $$b=C\left(2K_{AK}-K_{CK}^{\prime }\right)+e\left(K_{AK}+\left(K_{CK}^{\prime }-1\right)K_{CK}^{\prime }\right)-{PK}_{CK}^{\prime }\left(2K_{CK}^{\prime }-1\right)$$

(58) $$c=Ce\left(K_{CK}^{\prime }-2K_{AK}\right)-CK_{CK}^{\prime }P-C^{2}K_{AK}$$

(59) $$d=C^{2}eK_{AK}$$

Equation (55) is solved by using the Cardano formula and taking as a solution a root that is positive at positive *e* values (see Supplementary Materials, Equations (S1)–(S6)). The concentrations of ATP, ADP, AMP, and Cr can be calculated from the concentration of CrP using Equations (50)–(53).

In its final form, the mathematical model is a system of five differential equations (Equations (9), (22), (29), (37) and (44)). The concentrations of all metabolites were calculated from phase variables using Equations (26)–(29), (33)–(35), (41)–(43), (50)–(53) and (55).

### 4.5. Additional Assumptions

The model was investigated under the assumption that the non-phosphorylated form of GP (GPb) is inactive. Indeed, in resting muscle, GP is practically inactive [4,18,60]. In addition, the low rate of glycogen breakdown is compensated for by its synthesis [4,18,64,65]. Moreover, when contraction is activated, the main contribution to the GP reaction is made by the activated form of GP (GPa) [21,38,61,67].

The activities of GPa and ATP-consuming processes (ATPase) were changed as parameters. The activity of the ATPase in resting muscle was chosen to provide a steady state in the energy metabolism. Our estimation of the rate of ATP production in resting muscle, based on the rate of glycolysis [8,24,38,55], gives a value in the range of 8 to 58 mmol h^−1^ kg^−1^ tissue. In the model, we chose the value of 35 mM h^−1^ for the rate of ATP production (and ATP consumption) in resting muscle.

### 4.6. Equations for Enzymatic Reaction Rates

The expression for the rate of GPa reaction in the right part of Equation (22) was created based on data presented in [14,72,73]. The expressions for the rates of glycolytic reactions in the right parts of Equations (9), (22), (29), (37) and (44) and the values of most kinetic parameters were taken from [74], with slight modifications. Enzyme activity values in skeletal muscle were obtained from the literature (Table 3).

#### 4.6.1. Glycogen Phosphorylase a

GP catalyzes the reversible reaction of glycogen degradation:

$${\mathrm{glycogen}}_{n}\;+\;{\mathrm{PO}}_{4}\;⇄\;\mathrm{glucose}\text{-}1\text{-}\mathrm{phosphate}\;+\;{\mathrm{glycogen}}_{n-1}$$

GPa is an activated form of the enzyme that, contrary to the GPb form, does not exert any significant allosteric regulation [72]. It is permanently connected to the glycogen chain and so we assume that it is saturated with glycogen. Thus, the reaction becomes a simple one-substrate–one-product reaction. Assuming the fast binding/dissociation of PO_4_ and G1P compared to the enzymatic reaction rate, we get the following kinetic formula for the GPa reaction:

(60) $$V_{GPa}=\frac{A_{GPa}\;K_{G1P}\left(\left[PO_{4}\right]-\frac{\left[G1P\right]}{K_{eq}}\right)}{K_{G1P}K_{P}+\left[G1P\right]K_{P}+\left[PO_{4}\right]K_{G1P}}$$

Here, *A_GPa_* is GPa activity, which is varied in the model from 0 to 3000 mM h^−1^. *K_G_*_1*P*_ = 2.7 mM, *K_P_* = 4 mM [14], *K_eq_* = 0.33 [73].

#### 4.6.2. ATPase

We chose a simple linear approximation for the dependence of the total rate of ATP-consuming processes in muscle on [ATP]:

(61) $$V_{ATPase}=A_{ATPase}\left[ATP\right]$$

The value of ATPase activity (*A_ATPase_*) is a varying parameter in the model. For resting muscle, this parameter was set equal to 7.05 h^−1^, which provides an ATP consumption rate of 35 mM h^−1^ at [ATP] = 4.96 mM.

#### 4.6.3. HK

(62) $$V_{HK}=A_{HK}\frac{\frac{\left[ATP\right]}{K_{1}}}{1+\frac{\left[ATP\right]}{K_{1}}+\frac{\left[G6P\right]}{K_{2}}}$$

*A_HK_* = 100 mM h^−1^, *K*_1_ = 1 mM, *K*_2_ = 5.5∙10^−3^ mM. Since the glucose concentration in the model is assumed to be constant, the equation for the HK reaction rate does not contain a dependence on glucose.

#### 4.6.4. PFK

We extended the expression for the PFK reaction rate from [74] by including the dependence on the phosphate concentration in accordance to data from [75]:

(63) $$V_{PFK}=A_{PFK}\left(1+5\frac{\left[PO_{4}\right]}{\left[PO_{4}\right]+K_{6}}\;\right)\cdot \frac{\left[ATP\right]\left[F6P\right]}{\left(K_{2}+\left[ATP\right]\right)\left(K_{1}+\left[F6P\right]\right)}\cdot \frac{\frac{1}{1+\frac{\left[AMP\right]}{K_{3}}}\;+\;\frac{2\left[AMP\right]}{K_{3}+\left[AMP\right]}}{1+10^{8}\frac{{\left(1+\left[ATP\right]/K_{4}\right)}^{4}}{{\left(1+\frac{\left[AMP\right]}{K_{3}}\right)}^{4}{\left(1+\frac{\left[F6P\right]}{K_{5}}\right)}^{4}}}$$

*A_PFK_* = 3000 mM h^−1^, *K*_1_ = 0.1 mM, *K*_2_ = 2 mM, *K*_3_ = 0.01 mM, *K*_4_ = 0.195 mM, *K*_5_ = 3.7∙10^−4^ mM, *K*_6_ = 3.9 mM.

#### 4.6.5. GAPDH

(64) $$V_{GAPDH}=A_{GAPDH}\frac{\frac{\left[GAP\right]\left[NAD\right]\left[PO_{4}\right]\;-\;\frac{\left[1,3DPG\right]\left[NADH\right]}{K_{eq}}}{K_{1}K_{2}K_{3}}}{\left(1+\frac{\left[PO_{4}\right]}{K_{3}}\right)\left(1+\frac{\left[GAP\right]}{K_{1}}+\frac{\left[1,3DPG\right]}{K_{5}}\right)\left(1+\frac{\left[NAD\right]}{K_{2}}+\frac{\left[NADH\right]}{K_{6}}\right)}$$

*A_GAPDH_* = 35,000 mM h^−1^, *K*_1_ = 0.13 mM, *K*_2_ = 0.13 mM, *K*_3_ = 3.4 mM, *K_eq_* = 5∙10^−4^ mM^−1^, *K*_5_ = 1.25∙10^−2^ mM, *K*_6_ = 2∙10^−3^ mM. In this equation, the *K_eq_* value used in [74] was corrected using data from [76].

#### 4.6.6. PGK


(65) $$V_{PGK}=A_{PGK}\frac{\frac{\left(\left[1,3DPG\right]\left[ADP\right]\;-\;\left[3PG\right]\left[ATP\right]/K_{eq}\right)}{K_{1}K_{2}}}{1+\frac{\left[ATP\right]}{K_{5}}+\frac{\left[ADP\right]}{K_{2}}+C\frac{\left[1,3DPG\right]}{K_{1}}+D\frac{\left[3PG\right]}{K_{6}}}$$

$$C=\frac{K_{4}+\left[ADP\right]+\frac{\left[ATP\right]K_{4}}{K_{5}}}{K_{2}}$$

$$D=\frac{K_{7}+\left[ATP\right]+\frac{{\left[ADP\right]K}_{7}}{K_{2}}}{K_{5}}$$

*A_PGK_* = 22,000 mM h^−1^, *K*_1_ = 2.2∙10^−3^ mM, *K*_2_ = 0.14 mM, *K_eq_* = 1000, *K*_4_ = 0.3 mM, *K*_5_ = 0.27 mM, *K*_6_ = 1.4 mM, *K*_7_ = 0.4 mM. In this equation, the *K_eq_* value used in [74] was corrected using data from [77].

#### 4.6.7. PK

(66) $$V_{PK}=A_{PK}\frac{\frac{\left[PEP\right]\left[ADP\right]}{K_{1}K_{2}}}{1+\frac{\left[PEP\right]}{K_{1}}+\frac{\left[ADP\right]}{K_{2}}+\frac{\left[PEP\right]\left[ADP\right]}{K_{1}K_{2}}+\frac{\left[ATP\right]}{K_{3}}}$$

*A_PK_* = 20,000 mM h^−1^, *K*_1_ = 0.05 mM, *K*_2_ = 0.415 mM, *K*_3_ = 0.35 mM.

As mentioned above, the enzymatic reactions catalyzed by AK, CK, PGLM, GPI, ALD, TPI, PGM, and ENO were replaced in the model by the equilibrium ratios of corresponding metabolites. The values of the corresponding equilibrium constants, designated as *K_AK_*, *K*′*_CK_*, *K_PGLM_*, *K_GPI_*, *K_ALD_*, *K_TPI_*, *K_PGM_*, and *K_ENO_*, are shown in Table 3.

### 4.7. Calculation of the AK and CK Reaction Rates

The rate of ATP production in the AK reaction was calculated as

(67) $$V_{AK}=\frac{d\left[AMP\right]}{dt}$$

since AMP in our model is used only in the AK reaction.

The rate of ATP production in the CK reaction was calculated as

(68) $$V_{CK}=\frac{d\left[Cr\right]}{dt}$$

since Cr in our model is used only in the CK reaction.

The derivatives *d*[*AMP*]/*dt* and *d*[*Cr*]/*dt* are expressed in terms of the derivative of the model variable *de*/*dt* using the equations given in the Supplementary Materials (Equations (S7)–(S16)).

### 4.8. Calculation Methods

The kinetics of the mathematical model were calculated using the CVODE solver from the SUNDIALS library [78]. The steady states of the model were explored using the AUTO 2000 software [79] and the KINSOL solver from the SUNDIALS library [78]. In this work, we considered only stable steady states.

The source files for the model can be downloaded from https://doi.org/10.5281/zenodo.19565806 (accessed on 31 July 2026).

**Table 3:** The main model parameters.

Parameter	Unit	Model	Experiment	References and Comments
*A_ATPase_*	h^−1^	3.0–3000		This parameter varied in the model. In resting muscle, it was set equal to 7.05 h^−1^, which provides an ATP consumption rate in the model equal to 35 mM h^−1^ at [ATP] = 4.96 mM.
*A_GPa_*	mM h^−1^	0–3000	1600–6600	Experimental numbers show maximal GPa activity in muscles [21,23,80,81]. In the model, the parameter value varied from 0 to 3000 mM h^−1^.
*A_HK_*	mM h^−1^	100	27–180	[23,80,82,83,84]
*A_PFK_*	mM h^−1^	3000	54–5080	[55,80,85,86,87,88]
*A_GAPDH_*	mM h^−1^	35,000	4800–35,280	[81,85,89,90,91,92,93]
*A_PGK_*	mM h^−1^	22,000	11,220–60,600	[81,85,90,92]
*A_PK_*	mM h^−1^	20,000	7320–46,440	[81,85,90,94]
*P* = [*ATP*] + [*ADP*] + [*AMP*]	mM	5.4	4.0–6.2	[9,31,37,38,40,41,42,44]
*C* = [*Cr*] + [*CrP*]	mM	25	9.8–28.0	[31,38,42]
*K_AK_*		1	1	[74]
*K_ALD_*	mM	0.1	0.093–0.150	[95]
*K*′*_CK_*		5	4.6	[66]
*K_ENO_*		3.5	0.54–5.28	[95]
*K_GPI_*		0.45	0.45–0.67	[96]
*K_PGLM_*		17	17	[97]
*K_PGM_*		0.24	0.089–0.29	[96]
*K_TPI_*		0.45 ^a^	0.45 ^a^	[74]
[NAD]	mM	0.3	0.34–0.35	[98,99]
[NADH]	mM	0.002	0.0005	[99]
pH		7.1	7.0–7.1	[66,100]
*Q*	mM	32.9		The parameter value was calculated using Equation (2) and steady-state metabolite concentrations obtained in the model for resting muscle.

^a^ While the equilibrium constant for the TPI reaction measured in vitro with purified enzyme is close to the value of 0.045 [96], the [GAP]/[DAP] ratio (which corresponds to the equilibrium constant) measured in vivo in different mammalian cells and tissues is much greater and ranges from 0.08 to 0.57 [40,42,101,102,103,104,105]. It was shown that GAP can interact with the reaction mixture components and produce inactive forms in solution, which can affect the value of the measured equilibrium constant [106]. Thus, we suggest that the TPI reaction in cells is in equilibrium because of its high activity, but the [GAP]/[DAP] ratio is affected by the presence of the inactive GAP forms produced due to its interaction with intracellular components. We used the value of 0.45 as an effective value for the TPI reaction equilibrium constant in cells that takes into account GAP interaction with intracellular components.

## Figures and Tables

**Table 2:** Concentrations of orthophosphate, CrP, ATP, ADP, and AMP in resting skeletal muscle.

Variable	Unit	Model	Experiment	References
[PO_4_]	mM	4.56	0.58–11.7	[38,40,44]
[CrP]	mM	17.8	4.8–21.0	[9,31,37,38,41,42,44]
[ATP]	mM	4.96	4.0–6.2	[31,38,41]
[ADP]	mM	0.4	0.14–0.8	[9,31,37,38,40,42,44]
[AMP]	mM	0.033	0.014–0.1	[31,38,40,42,44]

## Data Availability

All data generated or analyzed during this study are included in the published article.

## Supplementary Materials

The following supporting information can be downloaded at https://www.mdpi.com/article/10.3390/ijms27167106/s1.
