# Supplementary material for: Activation of ATP Consumption Is Necessary to Stimulate Glycogenolysis in Skeletal Muscle
Source: Int J Mol Sci. 2026 Aug 8;27(16):7106. doi: 10.3390/ijms27167106 (PMC13513785; doi:10.3390/ijms27167106)
Supplement: Supplementary file 1 [file ijms-27-07106-s001.zip › ijms-4363656-supplementary.pdf]

Activation of ATP consumption is necessary to stimulate glycogenolysis in skeletal muscle.

Michael V. Martinov, Svetlana I. Sudarkina, Fazoil I. Ataullakhanov, Victor M. Vitvitsky

Supplementary materials

Applying the Cardano formula for solving the equation for creatine phosphate concentration.

We used the Cardano formula for solving cubic equation (equation (55) in the main text) and for the expression of [CrP] as a function of *e*:

$$[CrP] = \varphi + \beta - \frac{b}{3a} \quad \text{(S1)}$$

here

$$\beta = -\frac{p}{3\,\varphi} \quad \text{(S2)}$$

$$\varphi = \left(-\frac{q}{2} + \theta^{\frac{1}{2}}\right)^{\frac{1}{3}} \quad \text{(S3)}$$

$$\theta = \left(\frac{p}{3}\right)^{3} + \left(\frac{q}{2}\right)^{2} \quad \text{(S4)}$$

$$q = \frac{2b^3 - 9abc + 27a^2 d}{27a^3} \quad \text{(S5)}$$

$$p = \frac{3ac - b^2}{3a^2} \quad \text{(S6)}$$

The parameter φ is a real number at positive values of *e*. The parameter *p* is also a real number. Thus, we calculate β directly from the condition for root selection (S2).

Calculation of the contribution of AK and CK reactions to ATP production in the model.

Although in our model the AK and CK reactions are considered to be in equilibrium, their contribution to ATP production can be calculated from the following relationships, since AMP is produced only in the AK reaction and Cr is produced only in the CK reaction:

$$V_{AK} = \frac{d[AMP]}{dt} \tag{S7}$$

$$V_{CK} = \frac{d[Cr]}{dt} \tag{S8}$$

The derivatives *d[AMP]/dt* and *d[Cr]/dt* can be expressed in terms of the derivative of the phase variable of the model *de/dt*.

By differentiating with respect to time the system of algebraic equations (44 - 48) from the main text, we obtain a system of linear equations with respect to the derivatives *d[ATP]/dt*, *d[ADP]/dt*, *d[AMP]/dt*, *d[CrP]/dt*, and *d[Cr]/dt*:

$$\frac{de}{dt} = 2\frac{d[ATP]}{dt} + \frac{d[ADP]}{dt} + \frac{d[CrP]}{dt} \tag{S9}$$

$$K'_{CK}[ADP]\frac{d[CrP]}{dt} + K'_{CK}\frac{d[ADP]}{dt} = [ATP]\frac{d[Cr]}{dt} + [Cr]\frac{d[ATP]}{dt} \tag{S10}$$

$$2K_{AK}[ADP]\frac{d[ADP]}{dt} = [ATP]\frac{d[AMP]}{dt} + [AMP]\frac{d[ATP]}{dt} \tag{S11}$$

$$\frac{d[ATP]}{dt} + \frac{d[ADP]}{dt} + \frac{d[AMP]}{dt} = 0 \tag{S12}$$

$$\frac{d[Cr]}{dt} + \frac{d[CrP]}{dt} = 0 \tag{S13}$$

Solving this system with respect to derivatives, we obtain expressions for the rates of the AK and CK reactions:

$$\frac{d[AMP]}{dt} = V_{AK} = -\frac{de}{dt}\cdot\frac{(2K_{AK}[ADP]+[AMP])([ATP]+[ADP]K'_{CK})}{Z} \tag{S14}$$

$$\frac{d[Cr]}{dt} = V_{CK} = -\frac{de}{dt}\cdot\frac{[ATP][Cr]+2K_{AK}[ADP][Cr]+K'_{CK}[CrP]([ATP]-[AMP])}{Z} \tag{S15}$$

here

$$Z = [AMP][ATP] + [ATP]^2 + [ATP][Cr] + 4[ADP][ATP]K_{AK} + 2K_{AK}[ADP][Cr] + K'_{CK}([ADP][AMP] + [ADP][ATP] - [AMP][CrP] + [ATP][CrP]) + 4K_{AK}K'_{CK}[ADP]^2 \tag{S16}$$

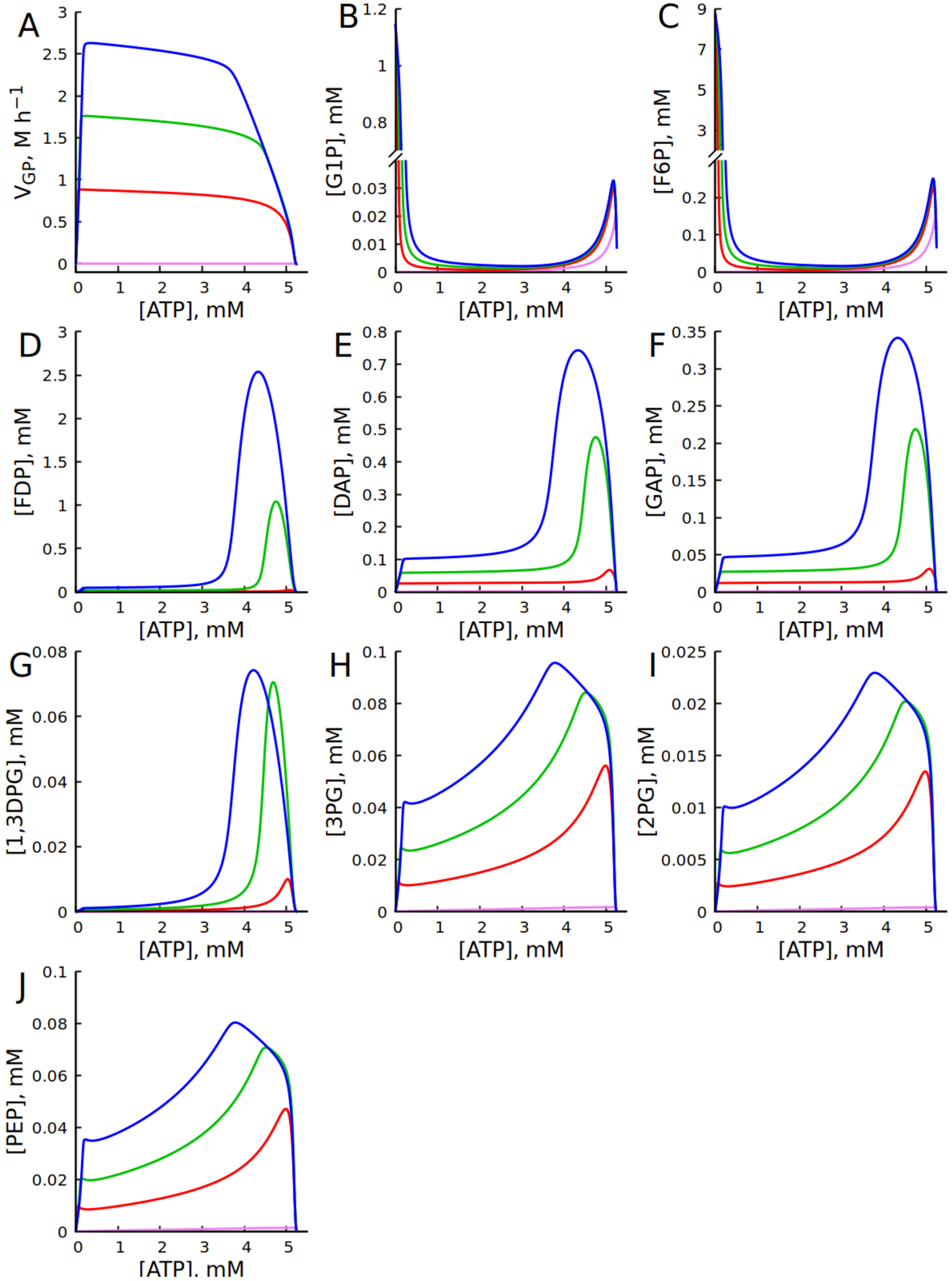

A
B
C
D
E
F
G
H
I
J
V_{GP}, M h^{-1}
[G1P], mM
[F6P], mM
[FDP], mM
[DAP], mM
[GAP], mM
[1,3DPG], mM
[3PG], mM
[2PG], mM
[PEP], mM
[ATP], mM

Figure S1. Effect of GPa activation on the steady-state characteristics of energy metabolism in skeletal muscle cells. A - The dependence of the steady-state rate of GP reaction on [ATP], B-K - The dependencies of the steady-state concentrations of glycolytic metabolites on [ATP]. The magenta, red, green, and blue lines represent data obtained at GPa activity of 0, 1000, 2000, and 3000 mM h^{-1} respectively.

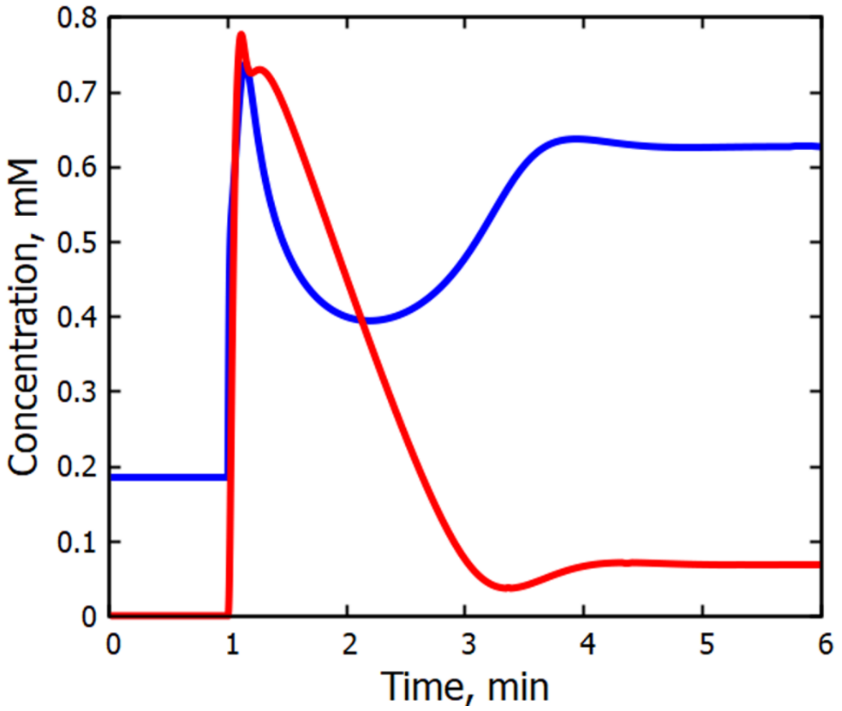

Figure S2. Kinetics of glycolytic metabolite concentration pools after GPa activation in resting skeletal muscle. GPa activity was increased from 0 to 3000 mM h^{-1} at 1 min. Blue line – [G1P]+[G6P]+[F6P], red line – [FDP]+[DAP]+[GAP].

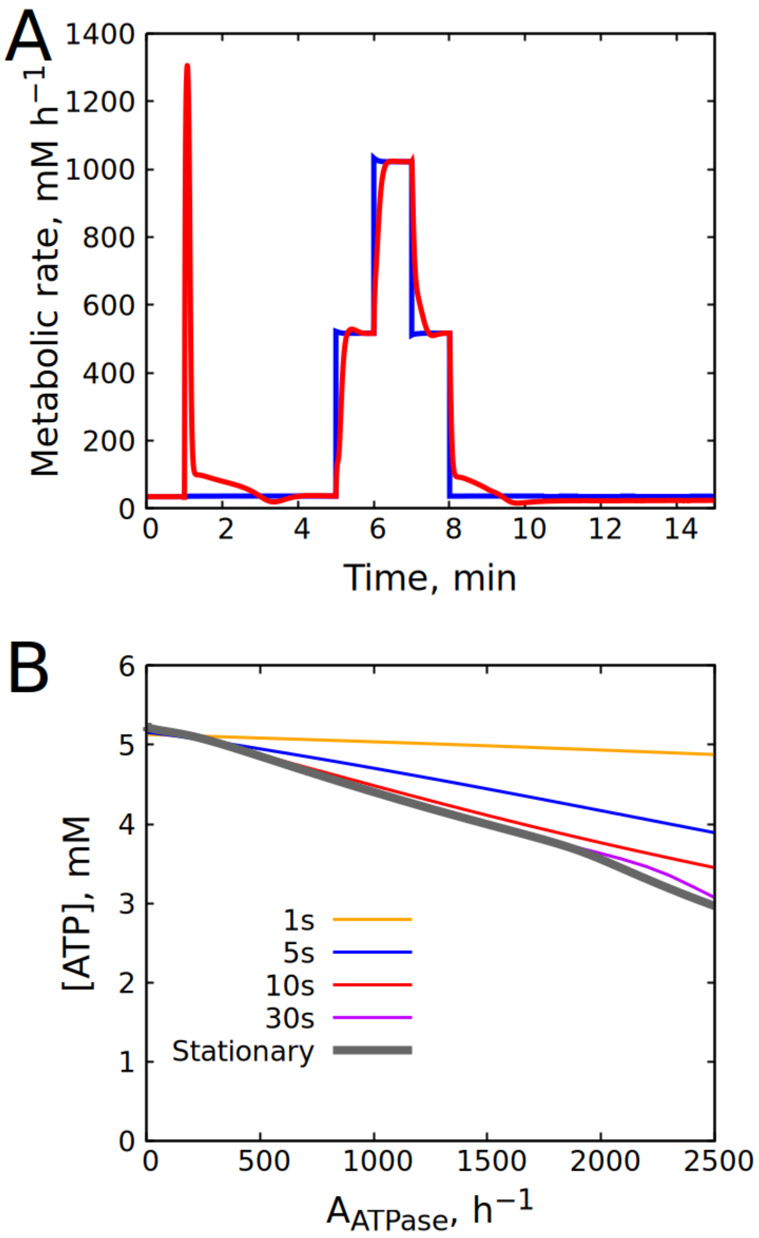

Figure S3. Energy metabolism in skeletal muscle operates near a steady state. A – Kinetics of the rates of consumption (blue line) and production (red line) of ATP in the model with changes in ATPase activity after activation of GPa. GPa activity was increased from 0 to 3000 mM h^{-1} at 1 min. ATPase activity was increased stepwise from 7.05 to 100 and 200 h^{-1} and then decreased to 100 and 7.05 h^{-1} at 5, 6, 7, and 8 min respectively. B – Dependence of ATP concentration on ATPase activity in the model at different time points after simultaneous activation of GPa and ATPase. GPa activity increased from 0 to 3000 mM h^{-1}. ATPase activity increased to a value equal to the value of the point coordinate on the X axis. The black line corresponds to a stationary dependence.

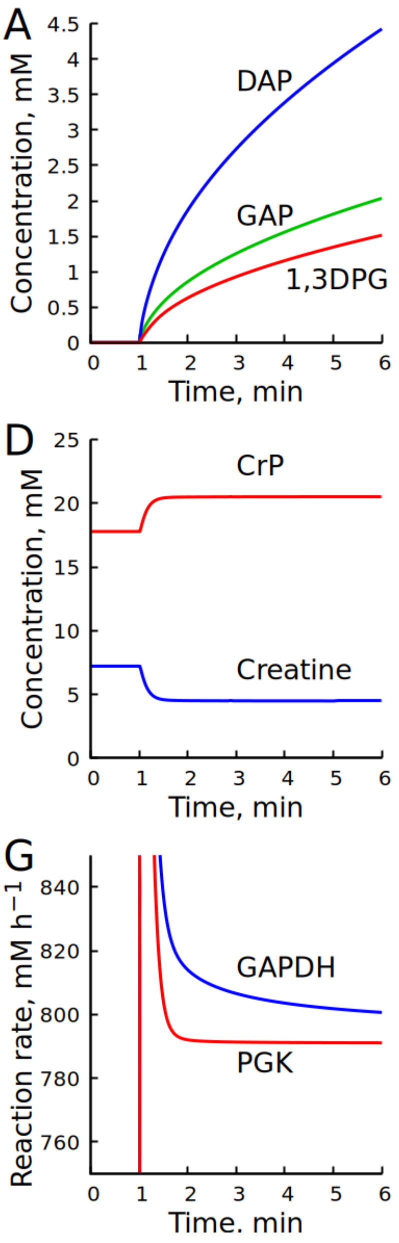

Figure S4. An activation of GPa in a resting muscle at constant orthophosphate concentration should cause a disappearance of the steady state with accumulation of metabolites that can cause a depletion of glycogen, osmotic swelling and destruction of muscle cells. A – Accumulation of DAP, GAP, and 1,3DPG; B-E – Kinetics of [G6P] and [F6P] (B), [3PG], [2PG], and [ENO] (C), [Cr] and [CrP] (D), [ATP], [ADP], and [AMP] (E); F – Kinetics of PFK and GAPDH reaction rates; G – The zoomed graph shows the difference between GAPDH and PGK reaction rates that cause accumulation of 1,3DPG. The PK and PGK reaction rates are equal to each other. The data were obtained in the model at constant $[PO_4]$ = 4.56 mM after an instant increase in the GPa activity up to 3000 mM h^{-1} at 1 min. Under this condition parameter *Q* in the model was not considered as a constant.

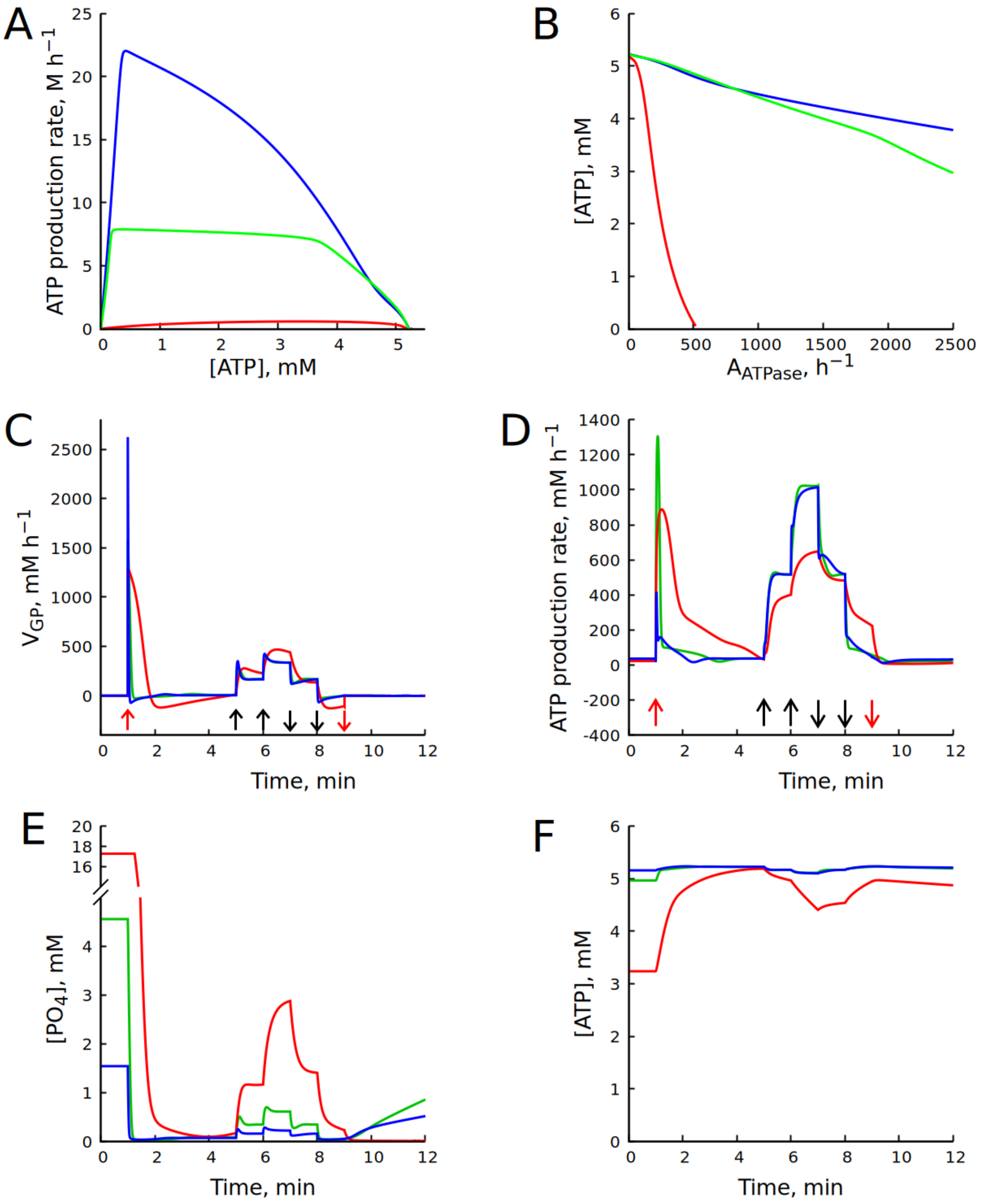

Figure S5. Dependence of the model results on the values of enzyme activity. A – Steady-state dependence of the rate of ATP production on the concentration of ATP, B – Steady-state dependence of ATP concentration on ATPase activity, C – Kinetics of the rate of the GP reaction during activation-deactivation of GPa and ATPase, D – Kinetics of the rate of ATP production

during activation-deactivation of GPa and ATPase, E - Kinetics of orthophosphate concentration during activation-deactivation of GPa and ATPase, F – Kinetics of ATP concentration during activation-deactivation of GPa and ATPase. The green line shows the results obtained with the enzyme activity values chosen for the model in Table 3. The red and blue lines show the results obtained, respectively, with the minimum and maximum set of enzyme activity values from Table 3. These values are equal to 1600, 27, 54, 4800, 11220, and 7320 mM h^{-1} for GPa, HK, PFK, GAPGH, PGK, and PK, respectively, for the minimum set and 10000, 180, 5080, 35280, 60600, and 46440 mM h^{-1} for the maximum set. Red arrows indicate the moments of activation (up) and deactivation (down) of GPa. At each set of activity values, GPa was activated to the corresponding maximum value. Black arrows indicate the moments of activation (up) and deactivation (down) of ATPase. ATPase activity was increased stepwise from 7.05 to 100 and 200 h^{-1} and then decreased to 100 and 7.05 h^{-1} for all values of other parameters.
